# Switching of electrochemical selectivity due to plasmonic field-induced dissociation

**DOI:** 10.1073/pnas.2404433121

**Published:** 2024-10-02

**Authors:** Francis M. Alcorn, Sajal Kumar Giri, Maya Chattoraj, Rachel Nixon, George C. Schatz, Prashant K. Jain

**Affiliations:** ^a^Department of Chemistry, University of Illinois Urbana-Champaign, Urbana, IL 61801; ^b^Department of Chemistry, Northwestern University, Evanston, IL 60208; ^c^Materials Research Laboratory, University of Illinois Urbana-Champaign, Urbana, IL 61801

**Keywords:** catalysis, electric field, plasmon, hot electron, energy

## Abstract

Electrochemical reactivity is known to be dictated by the structure and composition of the electrocatalyst–electrolyte interface. Here, we show that optically generated electric fields at this interface can influence electrochemical reactivity insofar as to completely switch reaction selectivity. We study an electrocatalyst composed of gold–copper alloy nanoparticles known to be active toward the reduction of CO_2_ to CO. However, under the action of highly localized electric fields generated by plasmonic excitation of the gold–copper alloy nanoparticles, water splitting becomes favored at the expense of CO_2_ reduction. Real-time time-dependent density functional tight binding calculations indicate that optically generated electric fields promote transient-hole-transfer-driven dissociation of the O─H bond of water preferentially over transient-electron-driven dissociation of the C─O bond of CO_2_. These results highlight the potential of optically generated electric fields for modulating pathways, switching reactivity on/off, and even directing outcomes.

## Significance

Quantized excitations produced by energetic stimuli such as plasmonic excitation potentially provide access to new chemical reaction and catalytic pathways. However, most examples in the areas of plasmon-induced chemistry and hot-electron catalysis are limited to rate enhancements. We demonstrate how photoexcitation of metal nanoparticle catalysts overturns reaction selectivity in a classic electrochemical reaction. Theoretical modeling elucidates that the selectivity change is the result of bond activation and dissociation induced by transient charge transfer under the action of optically generated electric fields. This work represents an important frontier for optical control of chemical and electrochemical reaction pathways on surfaces, the use of solar photons for chemical manufacturing, and insight into the role of electric fields in molecular activation and catalysis.

Plasmon-induced chemistry constitutes a remarkably interesting class of phenomena. Myriad reports have shown that optical excitation of localized surface plasmon resonances (LSPRs)—collective oscillations of charge carriers—in metal nanoparticles produces energetic species, induces energetically demanding chemical reactions, and enhances catalytic and electrocatalytic activity. Nevertheless, the mechanisms of these phenomena have not been fully elucidated, especially because of the confluence of multiple effects that can enhance chemical reactivity: generation of excited-state carriers by the relaxation of LSPRs (1–3), photothermal heating of the nanoparticle surface by nonradiative dissipation of the excited-state carriers (3–7), and intense electric near fields generated at the nanoparticle surface (3, 8–11). The relative contribution of the first two effects has been the subject of considerable debate and discussion in the community (4–6, 12), whereas the third effect has received less attention.

Here, we show that plasmonic excitation of a metal nanoparticle-based electrocatalyst leads to a complete switch in reaction selectivity under aqueous CO_2_ reduction reaction (CO_2_RR) conditions. We synthesize Au–Cu alloy nanoparticles and investigate the effect of plasmonic excitation on their electrocatalytic activity while properly accounting for the effect of photothermal heating. In addition to a nonthermal boost in the overall kinetics of electrochemical reactions on the nanoparticles, plasmonic excitation switched the selectivity of the electrocatalysis in favor of H_2_O splitting at the expense of CO_2_ reduction. Modeling by a real-time time-dependent density functional tight binding (RT-TD-DFTB) method shows that strong near-fields generated on the nanoparticle surface by plasmonic excitation selectively promote hole-driven dissociation of the O─H bond of H_2_O over electron-driven dissociation of the C─O of CO_2_. This effect induces a switch in selectivity in favor of H_2_ production over CO production. The near-field effect uncovered here advances our understanding of plasmon-induced chemistry and informs the design of plasmonic catalysts and processes.

## Materials and Methods

As a model system, we chose an alloy of Au and Cu. These coinage metals are not only plasmonic in the visible region (12–15) but are also the best known for their electrocatalytic CO_2_ reduction activity (16–19). Cu and Cu-rich Au–Cu alloy nanoparticles produce a variety of gas and liquid-phase products, including CO, H_2_, methane, ethylene, formate, acetate, and ethanol, with the product profile dependent on the applied potential (19). On the other hand, Au and Au-rich Au–Cu alloy nanoparticles generate CO and H_2_ as the major products (19). The simpler product profile of Au and Au-rich Au–Cu alloy nanoparticles is preferable for rigorous tracking of product selectivity and its dependence on plasmonic excitation. As an additional consideration, Au-rich Au–Cu alloy nanoparticles exhibit the highest mass activity for CO outperforming Au nanoparticles (19) thereby making the former best suited for reliable tracking of changes in product selectivity.

**Fig. 1.**
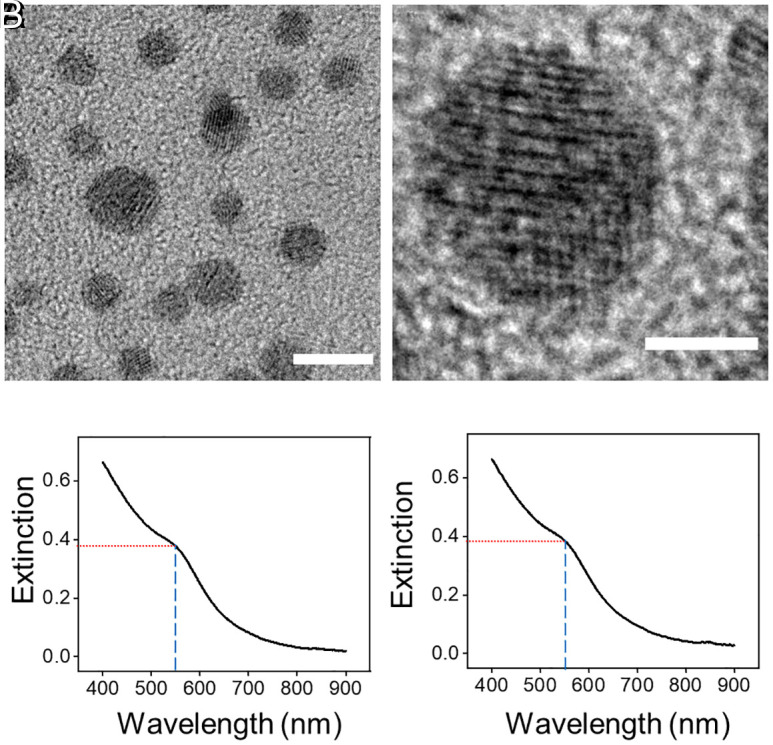
Structural and optical characteristics of Au–Cu nanoparticle catalysts. (*A* and *B*) High-resolution transmission electron microscopy (HRTEM) images of (*A*) multiple Au–Cu nanoparticles showing crystallinity; (*B*) an example showing multiple domains within a nanoparticle. [Scale bars are 5 nm and 2 nm in length in (*A*) and (*B*), respectively.] (*C* and *D*) Extinction spectrum of Au–Cu nanoparticles dispersed in hexane (*C*) acquired under an Ar atmosphere, and (*D*) after several hours of standing in air. Blue dashed lines indicate the approximate peak maximum wavelength of 550 nm for the LSPR peak and the red dashed lines indicate the extinction at this wavelength.

The Au–Cu alloy nanoparticles were synthesized using a seed-based method (20, 21). The mean diameter of these nanoparticles was 2.4 nm with a SD of 0.8 nm as measured by transmission electron microscopy (TEM) imaging (Fig. 1 *A* and *B* and *SI Appendix*, Fig. S1 *A* and *B*). X-ray diffraction (XRD) of these nanoparticles shows a structure with an FCC unit cell with a lattice constant of 3.93 Å (*SI Appendix*, Fig. S1*C*) consistent with an alloy of 68 mol % Au and 32 mol % Cu. Elemental analysis by X-ray fluorescence (XRF) spectroscopy shows a composition of 55 mol % Au and 45 mol % Cu. The discrepancy between XRD and XRF results suggests that the material contains a fraction of Cu in an amorphous or oxidized form.

Further, the Au–Cu alloy nanoparticles were plasmonic with an LSPR band centered around 550 nm (Fig. 1*C*). The nanoparticles were also resistant to oxidation in air as seen from the stability of the LSPR absorption (Fig. 1*D*) unlike monometallic Cu nanoparticles, which undergo oxidation when exposed to air resulting in a redshift of the LSPR of the Cu core due to the formation of an oxide shell (22–26).

By leveraging the LSPR absorption of the Au–Cu nanoparticles, we explored the effect of plasmonic excitation on electrocatalytic CO_2_RR on these nanoparticles in an aqueous environment. Working electrodes were prepared by depositing nanoparticles on glassy carbon electrodes (GCE) and subjecting them to electrochemical oxidation–reduction cycling to remove ligands and expose metallic active sites (*SI Appendix*, Fig. S2). We also determined by X-ray photoelectron spectroscopy (XPS) the composition of nanoparticles on the electrode following electrochemical cycling and found it to be 82 mol % Au and 18 mol % Cu (*SI Appendix*, Fig. S3) suggesting that Cu partially dissolved during electrochemical cycling; however, Au was maintained as both the primary plasmonic and CO_2_RR-active component.

**Fig. 2.**
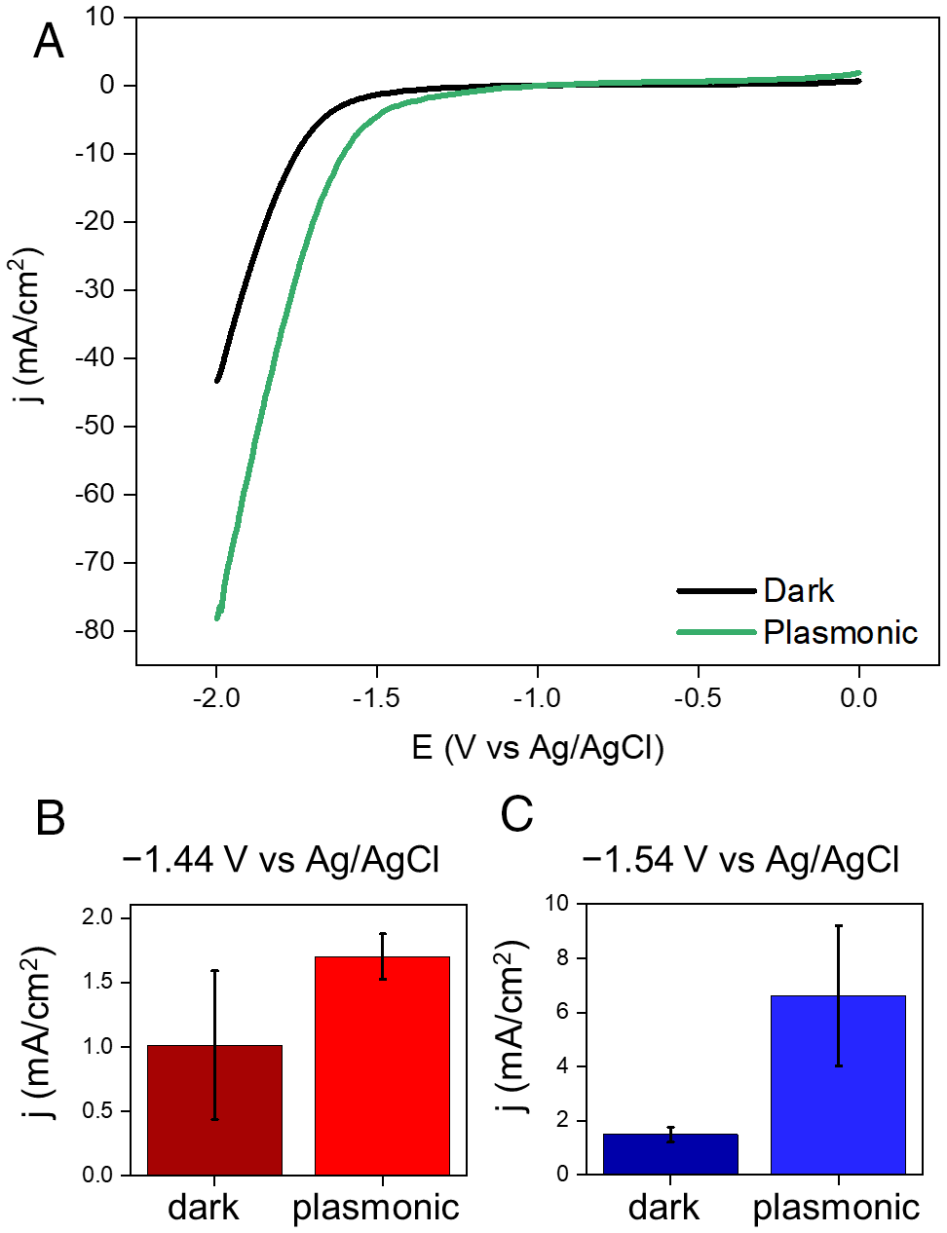
Nonthermal plasmonic enhancement of electrochemical reduction reactions. (*A*) Linear sweep voltammetry (LSV) of a Au–Cu nanoparticle-coated GCE in CO_2_-saturated 0.1 M K_2_SO_4_ from –2.0 to 0 V vs Ag/AgCl, sat. KCl at a scan rate of 50 mV/s acquired under plasmonic excitation achieved by 532 nm laser irradiation of a power of 1.45 W (green) and under dark conditions at an electrode surface temperature of 45 °C (black). The data used in the plot are a mean of 6 measurements on separate samples at each condition. The measured current was scaled by the ECSA to obtain the current density, which was then averaged. (*B* and *C*) Average current density in an 8 h CA scan of a Au–Cu nanoparticle-coated GCE in CO_2_-saturated 0.1 M K_2_SO_4_ at an applied potential of (*B*) –1.44 V and (*C*) –1.54 V vs Ag/AgCl, sat. KCl under plasmonic excitation achieved by 532 nm laser irradiation of a power of 1.45 W (light bars) and under dark conditions at an electrolyte temperature of 45 °C (dark bars). Each data point is a mean of three trials on separate samples; the error bar denotes the propagated SE.

Electrocatalysis, with and without plasmonic excitation (*SI Appendix*, Fig. S4), was studied at applied potentials of –1.44 V or –1.54 V vs Ag/AgCl, saturated (sat.) KCl in a CO_2_-saturated electrolyte consisting of 0.1 M K_2_SO_4_. The operating window of potentials is restricted by two considerations. A potential sufficiently more negative than the onset potential in the dark (Fig. 2*A*) is needed to ensure large enough rates of CO and H_2_ production for statistically reliable measurements of selectivity changes induced by plasmonic excitation. At the same time, we must avoid potentials that are too negative; because at considerably large overpotentials, H_2_ bubbles are produced at the electrode surface, which interfere with plasmonic excitation and mass transport and impede systematic measurements.

In plasmon-assisted electrocatalysis, the working electrode coated with the plasmonic nanoparticles was irradiated with a 532 nm laser with an incident power of 1.45 W and an irradiation spot 0.7 cm in diameter.

## Results and Discussion

Two main products were detected in the headspace of the electrochemical cell following 8 h of reaction. CO was produced by CO_2_ reduction as ascertained by ^13^C-labeling (*SI Appendix*, Fig. S5) and control experiments without CO_2_ (*SI Appendix*, Fig. S6). Only a minor rate of CO production was detected without the Au–Cu nanoparticles confirming that the nanoparticles were the active electrocatalysts (*SI Appendix*, Fig. S6). H_2_ generated by electrochemical reduction of water was the other major product.

To determine plasmonic effects, the results of plasmon-assisted electrocatalysis were compared with those of electrocatalysis on these working electrodes without any laser excitation (referred to here as “dark” electrocatalysis). The dark experiments were performed at an electrolyte temperature of 45 °C to match the highest measured surface temperature of the working electrode under plasmonic excitation. This accounts for the photothermal effect of plasmonic excitation (4–6) and ensures that any differences between plasmon-assisted and dark electrocatalysis could be attributed primarily to nonthermal photochemical effects resulting from plasmonic excitation. Second, as the electrochemically active surface area (ECSA) varies from one electrocatalyst sample to another, we scaled activity metrics (electrochemical current, rate of CO production, and rate of H_2_ production) measured in each trial by the ECSA of the sample used in that trial.

Under plasmonic excitation, the current density, i.e., current scaled by ECSA, was enhanced relative to dark conditions at all potentials more negative than –1 V vs Ag/AgCl, sat. KCl (Fig. 2*A*). This indicates nonthermal plasmonic enhancement of rates of electrochemical reduction reactions occurring on the nanoparticles. The average current density in an 8 h chronoamperometry (CA) scan was enhanced under plasmonic excitation by ~1.7× at –1.44 V and ~4× at –1.54 V vs Ag/AgCl, sat. KCl (Fig. 2 *B* and *C*).

**Fig. 3.**
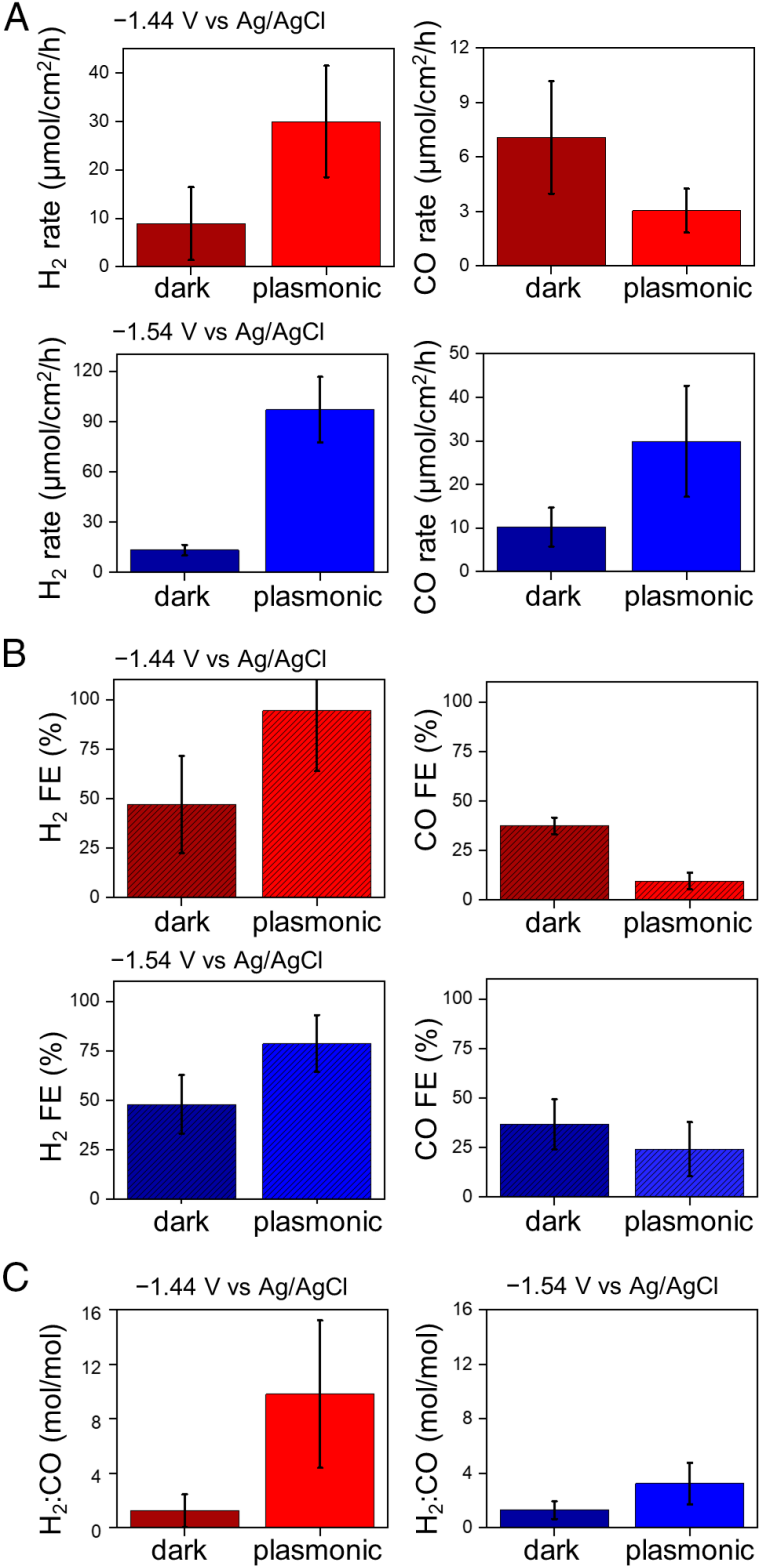
Plasmonic modulation of selectivity. (*A*) ECSA-scaled rate of H_2_ production (*Left*) and CO production (*Right*) in an 8 h reaction on Au–Cu nanoparticle-coated GCE at –1.44 V and –1.54 V vs Ag/AgCl, sat. KCl under plasmonic excitation achieved by 532 nm laser irradiation of a power of 1.45 W (light bars) and under dark conditions at an electrode surface temperature of 45 °C (dark bars). (*B*) FEs (%) of H_2_ production (*Left*) and CO production (*Right*) in an 8 h reaction on Au–Cu nanoparticle-coated GCE at –1.44 V and –1.54 V vs Ag/AgCl, sat. KCl under plasmonic excitation achieved by 532 nm laser irradiation of a power of 1.45 W (light bars) and under dark conditions at an electrode surface temperature of 45 °C (dark bars). FEs are provided and further analyzed in *SI Appendix*, Table S1. (*C*) Relative H_2_:CO yield, obtained by taking the ratio of the H_2_ production rate to the CO production rate, in an 8 h reaction on Au–Cu nanoparticle-coated GCE at –1.44 V (*Left*) and –1.54 V (*Right*) vs Ag/AgCl, sat. KCl under plasmonic excitation achieved by 532 nm laser irradiation of a power of 1.45 W (light bars) and under dark conditions at an electrolyte temperature of 45 °C (dark bars). Each data point is a mean of 3 trials on separate samples; the error bar denotes the propagated SE.

Since there are two major products, CO and H_2_, we were able to go beyond overall electrochemical rates and examine the effect of plasmonic excitation on the product branching. At both potentials applied in our study, the ECSA-scaled rate of H_2_ production was enhanced under plasmonic excitation (Fig. 3*A*), three-fold and seven-fold relative to dark conditions at –1.44 V and –1.54 V vs Ag/AgCl, sat. KCl, respectively. The ECSA-scaled rate of CO production was suppressed two-fold under plasmonic excitation at –1.44 V vs Ag/AgCl, sat. KCl (Fig. 3*A*) and enhanced three-fold under plasmonic excitation at –1.54 V vs Ag/AgCl, sat. KCl. An ordinary photothermal heating effect—for instance, via a decreased CO_2_ solubility in the electrolyte at elevated temperature—cannot account for the opposite trends observed at the two potentials.

Since the overall electrochemical charge consumption rate is always higher under plasmonic excitation (Fig. 2), it is more instructive to examine the Faradaic efficiency (FE). Plasmonic excitation boosts the FE of H_2_ production while suppressing the FE of CO production at both applied potentials (Fig. 3*B*). Thus, even though plasmonic excitation enhances charge harvesting, plasmonic excitation induces a greater fraction of the charge to be channeled toward H_2_ production and a smaller fraction toward CO production. This preference for the H_2_ production pathway is also evident in the larger relative H_2_:CO yield observed under plasmonic excitation at both applied potentials (Fig. 3*C*). In effect, plasmonic excitation promotes water splitting at the expense of CO_2_ reduction.

Thus, the excitation of LSPRs of Au–Cu nanoparticles switches their electrocatalytic selectivity. Plasmon-induced changes in reaction selectivity have been previously reported (27–32), although the mechanism is not always well resolved. Because we accounted for photothermal effects, the observed plasmon-induced modulation of selectivity is nonthermal.

**Fig. 4.**
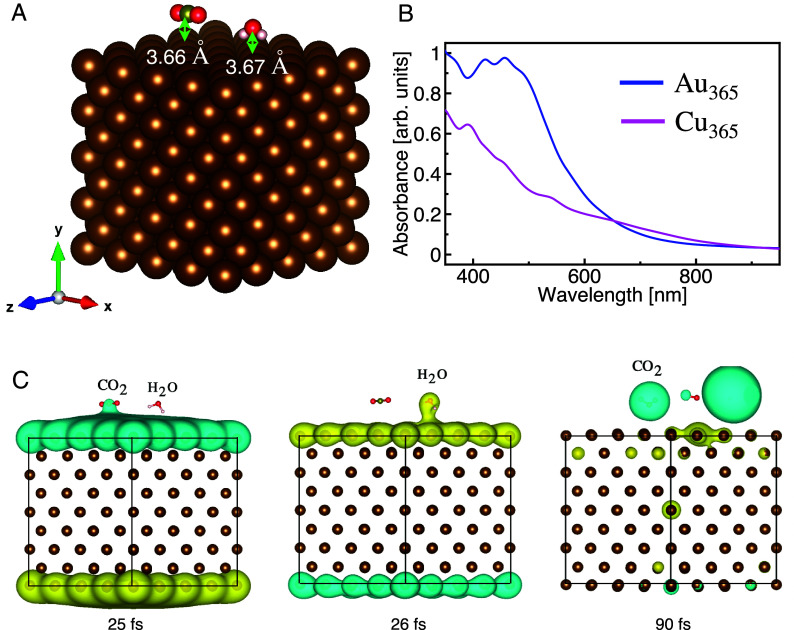
RT-TD-DFTB modeling of adsorbate photoactivation. (*A*) Optimized geometry of Au_365_CO_2_H_2_O, (*B*) calculated absorption spectra for Au_365_ and Cu_365_, and (*C*) map of the difference in charge density (yellow for positive and cyan for negative) for Au_365_CO_2_H_2_O with respect to the initial charge density at *t* = 0 when the system is driven by a Gaussian laser pulse (linearly polarized along the y-axis) that extends between [0, 50] fs, with parameters $\omega_{0}=2.5$ eV, and $E_{0}=1.5$ V/Å. Snapshots at 25 and 90 fs showing CO_2_ activation through transient electron transfer and snapshot at 26 fs showing H_2_O activation through transient hole transfer induced by the oscillating electric field. In (*A*), Au atoms are shown in brown, H in light pink, C in green, and O in red.

To elucidate the mechanism of nonthermal action, we performed calculations using the RT-TD-DFTB method implemented in the DFTB+ code (33, 34) to simulate the effect of plasmonic excitation on the activation of H_2_O and CO_2_ molecules on the surfaces of the nanoparticles. First, we optimized Au and Cu nanocubes each containing 365 atoms (with an overall charge of +1), which approximates the 2-nm diameter of the synthesized nanoparticles. Calculated absorption spectra for the bare Au and Cu nanoparticles are shown in Fig. 4*B*. A prominent plasmon peak appears for the Au nanoparticle at around 2.5 eV (496 nm), whereas for the Cu nanoparticle, the absorption spectrum is dominated by interband transitions, and the overall absorbance is smaller compared to the case of Au. As the nanoparticles under plasmon-assisted electrocatalysis conditions are 82 mol % Au in composition, our further calculations focused on pure Au nanoparticles. Cu was not included due to the difficulty of obtaining Slater–Koster parameters required to parameterize the Hamiltonians for a Au–Cu alloy. To generate geometries of nanoparticle–adsorbate complexes, we located H_2_O or CO_2_ on the flat surface of the nanoparticles and optimized the geometries of these adsorbates while keeping the atoms of the nanoparticle frozen. The distance between the O (C) atom and the nearest Au atom is about 3.67 Å (3.66 Å) for H_2_O (CO_2_). Both molecules are nearly charge-neutral in the adsorbed state. The optimized geometry for Au_365_CO_2_H_2_O is shown in Fig. 4*A*.

**Fig. 5.**
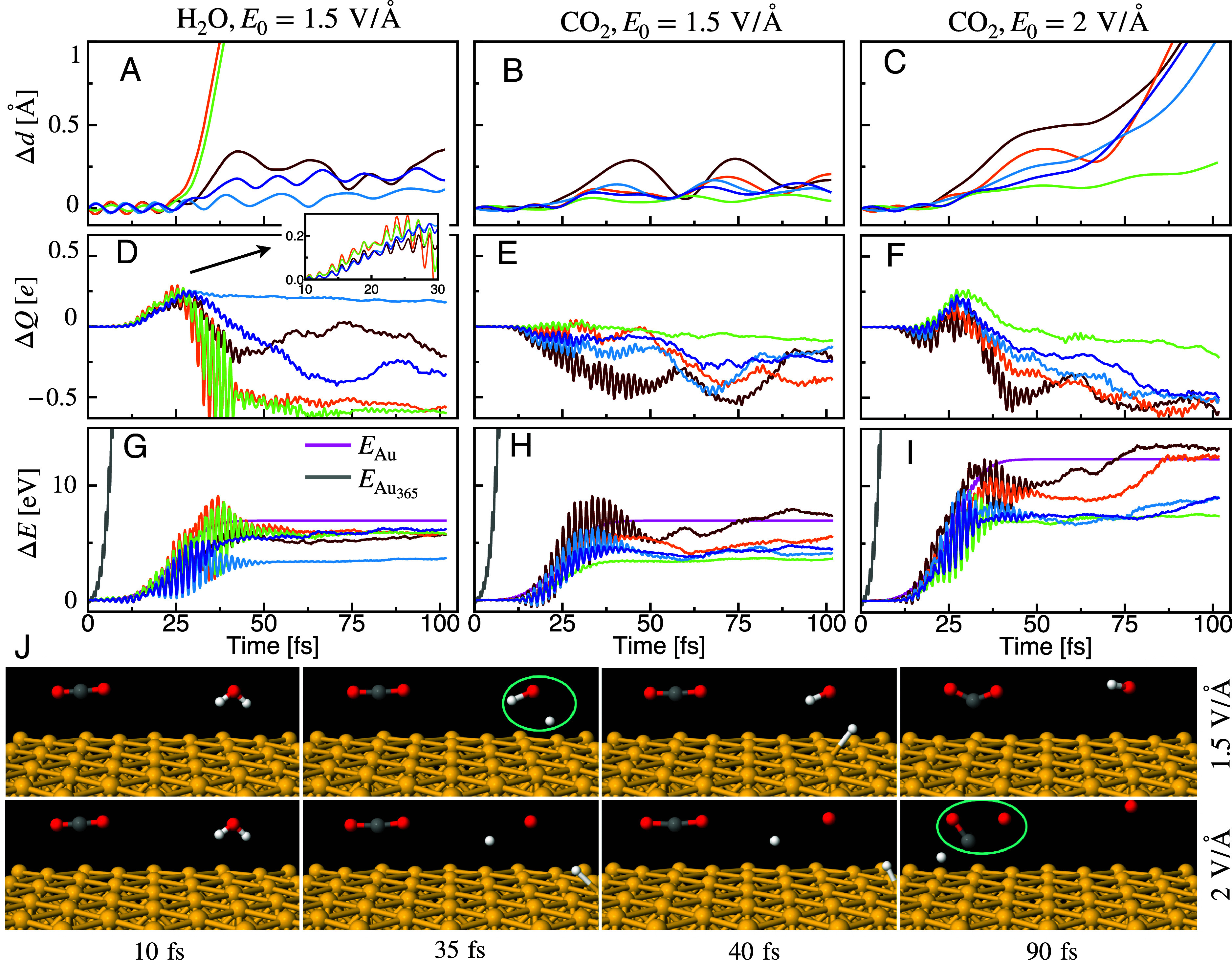
Dynamics of dissociation. Real-time trajectories (different replicas shown by different colors) for the (*A*) change in O─H bond distance Δd with *E*_0_ = 1.5 V/Å, (*B*) change in C─O bond distance Δd with *E*_0_ = 1.5 V/Å and (*C*) change in C─O bond distance Δd with *E*_0_ = 2 V/Å. Corresponding trajectories for the (*D*–*F*) change in molecular charge ΔQ and (*G*–*I*) change in molecular energy ΔE, with the same color code from panels *A*–*C*, respectively. The change is defined with respect to the initial value at *t* = 0 when a Gaussian laser pulse extending between [0, 50] fs and with a central frequency $\omega_{0}=2.5$ eV is applied to drive the system. In panel (*D*), an inset with a magnified view of the 10 to 30 fs range is provided. In (*G*–*I*), the trajectories for the change in energy ΔE for the entire nanoparticle and per Au atom are shown by gray and magenta lines, respectively. (*J*) Snapshots of molecular geometries along a trajectory (*Top row*) at *E*_0_ = 1.5 V/Å showing the dissociation of H_2_O and another trajectory (*Bottom row*) at *E*_0_ = 2 V/Å showing the dissociation of CO_2_. Examples of dissociated states are circled in green. Au atoms are shown in yellow, H in white, C in gray, and O in red.

Next, we study the response of the optimized Au_365_CO_2_H_2_O system to an incident optical field. The system is driven with a Gaussian laser pulse that extends between [0, 50] fs with a peak position at 25 fs and central frequency $\omega_{0}$ of 2.5 eV (*SI Appendix*, Fig. S7). Such pulsed excitation allows the study of ultrafast dynamics that underlies the photoactivity observed in experiments. The pulse is linearly polarized along the y-axis (orientation indicated in Fig. 4*A*). We use the Ehrenfest approximation to describe electron–nuclear dynamics, and for each choice of pulse parameters, several trajectories sampled from a 300 K canonical ensemble are calculated using the DFTB+ code. The real-time trajectories for the O─H (for H_2_O) and C─O (for CO_2_) bond distances (plotted in terms of $\Delta d=d\left(t\right)-d_{0}$ where $d_{0}$ is the initial bond distance) are shown in Fig. 5 *A*–*C*. It is important to note that isolated molecules (without the nanocube) do not dissociate even at a very high laser intensity (*SI Appendix*, Fig. S11). H_2_O dissociation has a threshold field amplitude $E_{0}$ of 1.5 V/Å on the Au nanocube surface, and it dissociates into H_(adsorbed)_ + ^.^OH. As seen from the trajectories (Fig. 5 *A* and *J**, Top* *row*), the molecule shows a coherent oscillation around the equilibrium geometry for an initial 25 to 30 fs followed by dissociation (orange and green trajectories in Fig. 5*A*) at around 35 fs. The generated H atom adsorbs to the surface of the particle after the dissociation at around 40 fs. However, CO_2_ does not dissociate at this field amplitude, only showing a coherent oscillation due to the vibrational motions of the molecule, and the average bond length is slightly elongated compared to the initial geometry (Fig. 5*B*). When the field amplitude is further increased to 2 V/Å, the CO_2_ molecule dissociates into O + CO as seen in Fig. 5 *C* and *J*. At this high field amplitude, both bonds in H_2_O dissociate. Interestingly, compared to the case for H_2_O, the dissociation is slower for CO_2_, which dissociates >30 fs after the interaction with the laser pulse has ended. After 40 fs, the CO_2_ transitions to a bent geometry, and subsequently the C─O bond dissociates at around 90 fs.

To obtain further insights into the dissociation mechanism (35), we calculated along the trajectories the change in the charge for the molecular fragment and the change in the energy for the nanoparticle and molecule fragments separately. Trajectories for the change in molecular charge ($\Delta Q=Q\left(t\right)-Q_{0}$ where $Q_{0}$ is the initial molecular charge) are shown in Fig. 5 *D*–*F*. As seen in Figs. 4*C* and 5*D*, there are hole transfers from the nanoparticle to H_2_O in the first 25 to 30 fs as seen by the molecule becoming partially positively charged ($Q_{\mathrm{molecule}}\approx +0.3e$). After the dissociation at ~35 fs, the additional charge is transferred back to the nanoparticle. This indicates that the dissociation of H_2_O is triggered by transient hole transfer. A similar hole-driven H_2_O dissociation mechanism is also observed in simulations with the Cu system (*SI Appendix*, Fig. S8).

In contrast, for the CO_2_, there is net electron transfer for the first few femtoseconds, followed by hole transfer, and after 35 fs, substantial electron transfer occurs, as seen by the molecule becoming partially negatively charged ($Q_{\mathrm{molecule}}\approx -0.6e$). Due to this charge transfer, the linear molecule transforms into a bent geometry, which eventually leads to the dissociation of a C─O bond at ~90 fs (Figs. 4*C* and 5 *E*–*F*). This means the CO_2_ dissociation is driven by transient electron transfer. The hole transfer for H_2_O and electron transfer for CO_2_ are clearly illustrated in Fig. 4*C*, indicating which orbitals on these molecules are active in the photoexcitation and involved in transient charge transfer from the nanoparticle to the adsorbed molecule. During the interaction of the system with the laser pulse, the energy of the nanoparticle rises rapidly (within 2 to 3 fs) compared to the molecular fragment; but following a small time-delay of around 10 fs, the energy of the molecular fragment increases due to the energy transfer mechanism (Fig. 5 *G*–*I*). H_2_O dissociates when its energy reaches a threshold value of around 3 to 6 eV, whereas CO_2_ dissociates at a much higher energy threshold of around 8 to 10 eV. Only the trajectories where threshold energies are reached involve dissociation, although all of them interact with identical laser fields. The transient-charge-transfer-mediated energy transfer from the nanoparticle to the adsorbed molecule is the driver for the dissociation as isolated molecules are nearly transparent to 532 nm excitation light and do not undergo dissociation (*SI Appendix*, Fig. S11).

To account for the effect of the external DC bias applied in the experiments, we study the dynamics of optical-pulse-induced dissociation of CO_2_ and H_2_O in the presence of a static field applied across the nanoparticle–adsorbate system. Even without the optical pulse, the static electric field polarizes the nanoparticle creating positive and negative poles along the polarization direction (*SI Appendix*, Fig. S9*A*). This also induces a charge on the adsorbate (*SI Appendix*, Fig. S9 *C* and *D*) with H_2_O acquiring a partial positive charge under negative static fields along the y-axis. On the other hand, CO_2_ acquires a partial positive or negative charge, respectively, under negative or positive static fields. Second, the Fermi energy of the nanoparticle increases with the applied field strength as expected (*SI Appendix*, Fig. S9*B*). We find that optical-pulse-induced O─H and C─O bond dissociation is enhanced in the presence of a static field. The fastest dissociation is observed for the O─H bond of H_2_O when the static field is negative and the induced charge on the H_2_O is positive (*SI Appendix*, Fig. S10).

Taken together, the RT-TD-DFTB simulations show that optically generated electric fields induce transient charge transfer across the metal nanoparticle–adsorbate interface (Fig. 4*C*), which in turn induces the dissociation of H_2_O and CO_2_ molecules at the surface of the nanoparticles. Transient hole-transfer-induced dissociation of the O─H bond of H_2_O is energetically more favorable than the transient electron-transfer-induced dissociation of the C─O bond of CO_2_. Analysis of the Kohn–Sham orbitals (36, 37) of the Au_365_CO_2_H_2_O system suggests that hole transfer from Au to the highest occupied molecular orbital (HOMO) of H_2_O requires an excitation energy of 2.91 eV, which is considerably lower than the energy of 4.92 eV required for electron transfer from Au to the unoccupied $\pi^{*}$ orbitals of CO_2_ (*SI Appendix*, Fig. S12). The preferential activation of H_2_O relative to CO_2_ is responsible for the H_2_-producing H_2_O splitting reaction being favored over the CO-producing CO_2_ reduction reaction under plasmonic excitation of the electrocatalytic nanoparticles.

## Conclusions

Plasmonic excitation of Au–Cu nanoparticles boosted the overall rate of electrochemical reactions catalyzed on the nanoparticles under aqueous CO_2_RR conditions. More crucially, plasmonic excitation switched the selectivity of the electrocatalysis in favor of H_2_O splitting at the expense of CO_2_ reduction. By properly accounting for photothermal heating, we confirm that the observed modulation of selectivity is a nonthermal effect. With the aid of simulations, we deduce that intense electric fields generated by plasmonic excitation induce transient charge transfer across the metal nanoparticle–adsorbate interface. Transient-hole-driven dissociation of H_2_O is energetically favored over the electron-driven dissociation of CO_2_, which causes a switch in selectivity in favor of H_2_ production over CO production. It is worth investigating whether this switch in selectivity is influenced by the nanoparticle alloy composition and/or the nature of excitation (plasmon-resonant vs interband excitation); these questions represent avenues for future work. The evolution and fate of the nanoparticle size and alloy composition in the plasmon-assisted electrocatalytic reaction is also worthy of a study. In a recent in situ TEM study of these nanoparticles supported on a carbon substrate and subjected to 532 nm laser irradiation, we found that plasmonic excitation triggers the Ostwald ripening of Au–Cu nanoparticles (38). Plasmon-induced Au nanoparticle coalescence has also been inferred by time-dependent dark-field scattering spectroscopy (39). The optical field-induced modulation of reactivity uncovered here furthers our mechanistic understanding of plasmon-induced chemistry and sets the stage for the use of optically generated electric fields for controlling chemical reactivity.

## Supplementary Material

Appendix 01 (PDF)

## Data Availability

All study data are included in the article and/or *SI Appendix*.
