# Supplementary material for: Switching of electrochemical selectivity due to plasmonic field-induced dissociation
Source: Proc Natl Acad Sci U S A. 2024 Oct 2;121(41):e2404433121. doi: 10.1073/pnas.2404433121 (PMC11474041; doi:10.1073/pnas.2404433121)
Supplement: Supplementary file 1 — Appendix 01 (PDF) [file pnas.2404433121.sapp.pdf]

SUPPORTING INFORMATION

Switching of electrochemical selectivity due to plasmonic field-induced dissociation

Francis M. Alcorn,[a] Sajal Kumar Giri,[b] Maya Chattoraj,[a] Rachel Nixon,[a] George Schatz,[b,] and Prashant K. Jain[a, c,]**

[a] Department of Chemistry, University of Illinois Urbana-Champaign, Urbana, IL 61801, USA

[b] Department of Chemistry, Northwestern University, Evanston, IL 60208, USA

[c] Materials Research Laboratory, University of Illinois Urbana-Champaign, Urbana, IL 61801, USA

***Corresponding authors:** Prof. Prashant K. Jain; Prof. George C. Schatz

E-mail address: jain@illinois.edu; g-schatz@northwestern.edu

Methods

Synthesis and characterization of Au–Cu nanoparticles

Au–Cu nanoparticles were synthesized by a seed-based method described by Li and coworkers[S1] using Au seed nanoparticles synthesized by a method described by Sun and coworkers.[S2] Briefly, Au seed nanoparticles were synthesized by injecting a reducing solution into a Au precursor salt solution under Ar at room temperature and stirring for 1 h. The Au precursor solution was prepared by dissolving 0.5 mmol $HAuCl_4$ (VWR, 99.99%) in a mixture of 20 mL of oleylamine (Sigma Aldrich, technical grade) and 20 mL of tetralin (Sigma Aldrich, 99%) and stirring under Ar for 15 min. The reducing solution was composed of 1 mmol tert-butylamine borane (97%, Fisher Scientific) in a mixture of 2 mL oleylamine and 2 mL tetralin. The synthesized Au seeds were isolated by addition of ethanol as an antisolvent at a 2:1 ratio of ethanol:Au seed solution and centrifuging the resulting mixture at relative centrifugal force (RCF) of 12,000 for 5 min. The supernatant solution was decanted off and the precipitate was then resuspended in hexane. This process was repeated two more times at a ~2:1 ratio of ethanol:hexane to thoroughly clean the nanoparticles. Then the Au seed nanoparticles were suspended in hexane and used for the alloying reaction, wherein a 3 mL dispersion of the seed nanoparticles (~0.43 mmol Au) in hexane was mixed with a Cu salt precursor solution. The Cu precursor salt solution consisted of 0.43 mmol $Cu(CH_3COO)_2$ (Acros Organics, 98%), 0.43 mL of oleic acid (Alfa Aesar, 90%), and 1.92 mL of tri-n-octylamine (Sigma Aldrich, 98%). The Cu precursor solution contained in a round-bottom flask was heated under Ar to a temperature of 70 °C using a sand bath, and the Au seed solution was injected into the flask. Hexane from the Au seed solution was evaporated, following which the temperature was increased to 120 °C and maintained at that value for 15 min. Then the temperature was increased to 280 °C and maintained at that value for 80 min. Thereafter, the reaction mixture was cooled to room temperature. All steps were performed under Ar. These nanoparticles were isolated by precipitating them by adding ethanol and centrifuging the resulting mixture at 12,000 RCF for 5 min. The precipitate was then resuspended in ~6 mL of hexane in an inert atmosphere of a glovebox.

This suspension was stored in the glovebox but eventually removed from the glovebox for characterization and studies.

The structure, composition, and optical properties of the synthesized Au–Cu nanoparticles were characterized. TEM imaging of the nanoparticles was performed using a Hitachi H-9500 instrument operating at an accelerating voltage of 300 kV. Two microliters of nanoparticles suspended in hexane were dropcasted on a TEM grid and allowed to air dry for these analyses. Images were acquired in Digital Micrograph and then converted to tag image file format (TIFF) for analysis of nanoparticle sizes in ImageJ (Fig. S1). For size analysis, several TEM images with many individually separated nanoparticles were selected. Each image was subjected to fast-Fourier transform (FFT) filtering and thresholding in ImageJ. FFT filtering was done to remove features smaller than 30 pixels in size, and an 8-bit threshold value of 102 was used. The areas of nanoparticles in the thresholded images were then measured in ImageJ using the 'Analyze Particles' tool. The areas were converted into diameters by assuming the nanoparticles were spherical in shape, i.e., diameter = $2 \times (\text{area}/\pi)^{1/2}$.

Powder XRD (Fig. S1) was performed using a Rigaku Miniflex X-ray diffractometer, from 2Θ = 25 to 90°, with 0.02° steps and 2 s integration per step. The XRD pattern was refined using PowderCell.

Elemental composition was determined by XRF under a He atmosphere using a Shimadzu EDX-7000 X-ray fluorescence spectrometer. Cu and Au amounts were quantified by integrating over X-ray energies of 7.84–8.24 keV and 9.48–9.98 keV, respectively, with 200 s integration times.

Visible-range extinction spectrophotometry was performed using a Shimadzu UV-3600 spectrophotometer. Spectra were acquired from 400 to 900 nm. Several spectra of a dispersion of the nanoparticles in hexane were acquired. The first (Fig. 1c) was an extinction spectrum of the dispersion in a sealed cuvette just after removal from an Ar-filled glovebox. Then the cap of the cuvette was removed, the dispersion was exposed to air, and several more spectra were acquired during the course of this air exposure, including one after ~6 h of standing in air (Fig. 1d).

Electrocatalytic CO_2RR experiments

Electrocatalytic CO_2RR experiments were performed using BioLogic SP-200 and CHI 6500A potentiostats. The working electrode was a glassy carbon electrode (GCE) coated with the Au–Cu alloy nanoparticles. The reference electrode was a Ag/AgCl saturated (sat.) KCl electrode. The counter electrode was a graphite rod. Prior to coating with nanoparticles, the GCE (L-type, 5 mm diameter) was polished with alumina slurries (0.3 and 0.05 μm size). Then a 25 μL dispersion of Au–Cu nanoparticles in hexane was dropcasted on it. To clean the nanoparticle surfaces and remove ligands, the nanoparticles deposited on the GCE were subjected to cyclic electrochemical oxidation–reduction. Specifically, cyclic voltammetry (CV) was performed wherein the potential was swept from 0 V to 1.0 V and then 60 CV scans were conducted from 1.0 V to –0.5 V vs Ag/AgCl, sat. KCl and back at a scan rate of 50 mV/s in 1 M KOH (Alfa Aesar, 85%) in deionized water (18.2 MΩ resistance) with a graphite rod counter electrode. The CV scans measured were used to determine the electrochemical surface area of the nanoparticles on the working electrode (Fig. S2) using a method previously reported by our group.[S3] Briefly, the peak in the final CV scan corresponding to reduction of the metal oxide layer formed on the surface of the nanoparticles at positive potentials in the scan was integrated in Origin and the resulting value was divided by the scan rate to yield the charge consumed in the reduction reaction. In redox cycling experiments conducted using the CHI potentiostat, CV scans beyond the 22nd cycle did not get recorded. Nevertheless, in every case, the last recorded scan was used for the analysis of ECSA. By dividing this consumed charge by the known charge required for reduction per unit area of a monolayer of Au oxide (0.39 mC/cm^2), we obtained the electrochemically active surface area. Although we did not consider the Cu in this analysis, this is justified because the post-cycling nanoparticles were primarily comprised of Au as determined by XPS analysis (Fig. S3).

CO_2RR studies were performed in an H-type two-compartment glass electrochemical cell (Fig. S4) with Teflon screw caps. Both sides were filled with 50 mL of 0.1 M K_2SO_4 (Sigma Aldrich, >99.0%) in deionized water (18.2 MΩ resistance). The pH of the catholyte was measured at the end of CO_2RR

experiments to be 4.9, on average. The two compartments were separated by a Nafion membrane (Nafion 212, Fuel Cell Store). To prevent leaking of the electrolyte, the junction between the two compartments was tightly clamped. The working and reference electrodes were immersed in the catholyte through two threaded ports in the Teflon screw cap. The ports were made airtight using Viton O-rings and hollow plastic screws that were tightly screwed into the threaded openings around the electrodes. Into a third port, a septum (Agilent, 5183-4757) was added, which was also made airtight by tightly screwing in a hollow screw. The Teflon screw cap, thus loaded with the working electrode, reference electrode, and a septum, was then tightened onto the cathodic compartment of the cell with a strap wrench. We sealed the brim of the cap with electrical tape. The graphite rod counter electrode was immersed in the anolyte and was left open to air. Prior to each CO_2RR experiment, the cathodic compartment, i.e., the compartment with the working electrode, was tightly sealed and the catholyte was saturated with CO_2 by bubbling CO_2 (Ultrahigh purity, AirGas) using a needle inserted through the septum. The bubbling was carried out for 30 min at a flow rate of 30 ± 5 mL/min as measured by an analog flow meter. After saturation with CO_2, 400 μL of the headspace of the cathodic compartment was drawn through the septum using an air-tight syringe and injected into an Agilent 7820A gas chromatograph (GC) equipped with a thermal-conductivity detector (TCD) for analysis of the initial gas composition of the headspace.

The cell was then subjected to either laser excitation for plasmon-assisted electrocatalysis or heating using a water bath for dark electrocatalysis. Plasmonic excitation was achieved using a 532 nm diode-pumped solid-state (DPSS) laser (CST-H-532NM-1500 MW) with a power of 1.45 ± 0.05 W. The laser beam was directed through the glass wall of the cathodic compartment onto the nanoparticle-coated GCE forming an irradiation spot 0.7 cm in diameter. In dark experiments, the cell was heated by submerging it in a heated and stirred water bath such that the temperature of the catholyte was maintained at 45 °C as measured by a thermocouple probe, which controlled the heating rate of the hotplate (VWR) used for heating the water bath. The electrochemical cell was allowed to sit for 1 h under these conditions to reach thermal equilibrium before electrocatalysis. We chose a temperature of 45 °C for these dark experiments

as it corresponds to the highest surface temperature of the nanoparticle-coated working electrode under plasmonic excitation as determined by a method developed in our laboratory.[S4] For the latter measurement, the nanoparticle-coated working electrode was immersed in the electrolyte and subjected to laser irradiation conditions similar to those used in plasmon-assisted electrocatalytic CO_2RR experiments. Irradiation was continued for >1 h to allow the electrode surface to reach its steady-state temperature under laser excitation. Then the laser beam was blocked, and the temperature of the electrode surface was measured over time by contacting a thermocouple probe. The plot of the temperature vs time in the course of this cooling was fit to Newton's cooling law formula. By extrapolating the fit to t = 0 s, i.e., the instant when the laser was blocked, we determined the electrode surface temperature under plasmonic excitation to be 39–45 °C. Therefore, dark experiments were carried out with the electrolyte temperature set to 45 °C.

Three identical trials were performed for each set of conditions: dark and plasmon-assisted electrocatalytic CO_2RR at two applied potentials: –1.44 or –1.54 V vs Ag/AgCl, sat. KCl. This amounted to twelve experiments in total. Each electrocatalytic CO_2RR experiment comprised of linear scanning voltammetry (LSV) from –2 to 0 V vs Ag/AgCl, sat. KCl at a scan rate of 50 mV/s, followed by 8 h of chronoamperometry (CA) at a constant potential of either –1.44 or –1.54 V vs Ag/AgCl, sat. KCl, and finally another round of LSV. The final LSV scan led to the data presented in Fig. 2a. The current vs. time plot measured over 8 h of CA was integrated to yield the consumed charge. By dividing the consumed charge by the total time (8 x 3600 s), we obtained the average current. By dividing the average current by the ECSA, we obtained the current density. The mean value of the current density over three trials corresponds to the data in Fig. 2b and c.

Gases in the headspace of the cathodic compartment were analyzed before and after the electrocatalytic CO_2RR by GC-TCD. For this analysis, 400 μL of the headspace was drawn from the cathodic compartment and injected into the GC-TCD instrument. Chromatography parameters were as follows: purge packed inlet heated to 150 °C, an Agilent G3591-80064 packed stainless-steel column, He carrier gas flowing at 35 mL/min, oven temperature of 100 °C, and a TCD temperature of 200 °C. Retention

times for H_2 and CO were 0.6 and 4.1 min, respectively. Reference chromatograms and calibration curves for H_2 and CO were generated by injecting H_2 and CO standards into the GC-TCD. An H_2 standard was produced by purging a septum-sealed vial with 5% H_2 in N_2. A specific volume of the vial headspace was extracted through the septum under continuous purging and injected into the GC-TCD. The headspace was assumed to be at atmospheric pressure amounting to a H_2 partial pressure of 0.05 atm. A series of different injection volumes were subjected to GC-TCD analysis. By plotting the moles of H_2 injected (estimated from the injection volume and partial pressure using the ideal gas law) against the integrated area of the H_2 peak in the chromatogram, we generated a calibration curve for H_2. Integrated peak areas were measured by integration in Origin. The calibration curve was fit to a zero-intercept straight line and the slope was used as a calibration constant. By multiplying the measured area of the H_2 GC peak by this calibration constant and the ratio of the headspace volume-to-injection volume, we obtain the moles of H_2 present in the headspace.

A CO standard was produced by reacting a known volume of oxalyl chloride (Thermo scientific, 98%) with an excess of aqueous hydroxide in a septum-capped vial.[S5] By assuming a stoichiometric reaction, i.e., 1 molecule of CO per molecule of oxalyl chloride, we estimated the moles of CO present in the headspace. A specific volume of the headspace of this vial was injected into the GC-TCD. By plotting the molar amount of CO in this injected volume (estimated by assuming a uniform concentration of CO in the sealed vial) against the integrated area of the CO peak in the chromatogram, we generated a calibration curve for CO. Integrated peak areas were measured by integration in Origin. The calibration curve was fit to a zero-intercept straight line and the slope was used as a calibration constant. By multiplying the measured area of the CO peak by this calibration constant and the ratio of the headspace volume to injection-volume, we obtain the moles of CO present in the headspace.

Faradaic efficiencies (FEs) were estimated by converting the moles of H_2 and CO produced in each 8 h CA experiment to the partial charge consumed by each product. This conversion was performed by multiplying the amount of each product by 2, which is the number of electrons required to produce either

CO or H_2 and by Faraday's constant. The partial charge for each product was then divided by total charge consumed in the 8 h CA experiment calculated by integration of the current vs time plot. The ratio obtained was then converted to a percentage. The calculation of FEs used mean values of the partial consumed charge and total consumed charge across three identical trials on separate samples for each data set. The error in the calculated FE was estimated by propagating the standard errors of these mean values *via* quadrature.

Between separate experiments, the GCE was cleaned to remove nanoparticles. This was done by rinsing the GCE with hexane and DI water and then soaking in aqua regia for at least 45 min.

Composition of electrocatalytically active nanoparticles

Although the chemical composition of the as-synthesized Au–Cu nanoparticles was characterized by XRD and XRF, the composition of the nanoparticles involved in the CO_2RR electrocatalysis is not necessarily the same due to chemical changes occurring under electrochemical conditions, especially the deposition on the GCE, exposure to the electrolyte, and oxidation–reduction cycling in the electrolyte. Therefore, we characterized using XPS the chemical composition of the nanoparticles that had undergone electrochemical processing and of nanoparticles that had not been subjected to these steps. To this end, a dispersion of Au–Cu nanoparticles in hexane was dropcasted onto two individual polished glassy carbon plates (GCPs) followed by drying overnight. One of these nanoparticle-coated GCPs was then subjected to electrochemical oxidation–reduction cycling while the other was left undisturbed. The former Au–Cu-nanoparticle-coated GCP was immersed along with a double-junction 3 M KCl Ag/AgCl reference electrode and a graphite rod counter electrode in a solution of 1 M KOH in deionized water. With the Au–Cu-nanoparticle-coated GCP serving as the working electrode, the potential was first swept from 0 V to 1.0 V and then 60 CV scans were conducted by scanning the potential from 1.0 V to –0.5 V vs Ag/AgCl, 3 M KCl and back at a scan rate of 50 mV/s. Following cycling, the Au–Cu nanoparticle-coated GCP was removed from the electrolyte, allowed to dry for 1 h, rinsed with water, and then dried in a vacuum desiccator overnight prior to XPS analysis of the GCP-supported Au–Cu nanoparticles (Fig. S3). The undisturbed Au–Cu-nanoparticle-coated GCP was also rinsed with water for consistency and allowed to

dry overnight prior to XPS analysis. XPS was performed on a Kratos Axis Supra$^+$ X-ray photoelectron spectrometer with an Al Kα anode X-ray source (1486.69 eV) at a power of 75 W (5 mA). A survey spectrum, high-resolution spectra in the Au 4f, Cu 2p, O 1s, and C 1s regions, and Auger spectra in the Cu LMM region were acquired. Prior to plotting and analysis, binding energies were referenced to the C 1s binding energy, which was assigned a value of 284.8 eV corresponding to aliphatic C–C bonds. Spectra were analyzed in CasaXPS software.

Control experiments

Several control experiments were performed (Fig. S5 and S6). These included an experiment without nanoparticles on the working electrode, an experiment without CO_2 in the cathodic compartment, and an experiment with the catholyte saturated with $^{13}CO_2$ instead of $^{12}CO_2$. In these control experiments, unless otherwise noted, all other procedures, parameters, and conditions were maintained the same as the plasmon-assisted electrocatalytic CO_2RR experiments performed at –1.54 V vs Ag/AgCl, sat. KCl. We performed three identical trials of the control experiment without nanoparticles. The GCE was thoroughly cleaned by polishing and prolonged soaking in aqua regia for several hours prior to this set of trials. No nanoparticles were deposited onto this cleaned GCE, but prior to each electrocatalytic experiment, the GCE was subjected to 30 cycles of CV scans in 1 M KOH. We performed three identical trials of the control experiment without CO_2. In each trial, the catholyte was saturated with N_2 by bubbling N_2 for 30 min at a flow rate of 30 ± 5 mL/min. For the ^{13}C-labeling experiment, which was undertaken to verify if CO detected in the headspace after electrocatalysis is indeed a product of CO_2RR, the catholyte was saturated with $^{13}CO_2$ by bubbling $^{13}CO_2$ (Aldrich, 99% ^{13}C) for 30 min at an unknown flow rate. Following the electrocatalysis, 2 mL of the gaseous headspace was extracted from the cathodic compartment and injected into a septum-capped vial (~20 mL). The gaseous contents of the vial were analyzed by GC–mass spectrometry (GC–MS) at the Metabolomics Core Facility of the Roy J. Carver Biotechnology Center (Fig. S6).

Real-time time-dependent density functional tight binding (RT-TD-DFTB) calculations

Recent papers[S6,S7] detail the DFTB method. This method uses parametrized Hamiltonians that involve Slater-Koster parameters. The parameters were downloaded from the *dftb.org* site. We used *auorg*/*auorg-1-1* parameters for Au- and *matsci*/*matsci-0-3* parameters for Cu-containing metal–molecule systems. We investigated the $Au_{365}CO_2H_2O$ and $Cu_{365}H_2O$ systems. We did not include CO_2 in the latter case as Cu–C parameters are not currently available.

The real-time method for DFTB was described recently by Sánchez and coworkers.[S7] The single-particle density matrix $\rho(t)$ evolves over time with an initial condition given by the ground-state Hamiltonian H_0, overlap matrix *S*, and density matrix ρ_0. We use the Ehrenfest method to incorporate nonadiabatic dynamics permitting energy exchange between electronic and nuclear degrees of freedom and propagate the density matrix solving the Liouville-von Neumann equation:

$$\dot{\rho} = \mathrm{i}\rho\left(H + \mathrm{i}D^{\dagger}\right)S^{-1} - \mathrm{i}\, S^{-1}(H - \mathrm{i}D)\rho \tag{1}$$

with overlap matrix *S* (S^{-1} is its inverse), Hamiltonian matrix *H*, and the nonadiabatic-coupling matrix *D* with elements $D_{\mu\nu} = \langle\phi_\mu|\dot{\phi}_\nu\rangle$ with localized atomic orbitals $|\phi\rangle$. The Ehrenfest method for plasmonic systems should be a suitable choice as ρ involves a high density of excited states.[S8,S9] Also, the computational effort associated with generating the Born-Oppenheimer (BO) surfaces is substantially higher for dynamics based on BO surfaces. Note that the above equation has two parts, pure electronic (through the Hamiltonian *H*) and coupling between electronic and nuclear degrees of freedoms (through the nonadiabatic coupling *D*). When the nonadiabatic coupling term vanishes—which is the case when the atoms are at rest, as the coupling depends on the time derivative of the localized atomic orbitals—the equation of motion becomes purely electronic. The Hamiltonian *H* contains the interaction between field and matter through a dipole coupling term $\mu E(t)$, where μ is the dipole moment and $E(t)$ is the time-dependent external electric field.

An equation of motion for the force that the electronic states exert on the nuclei is obtained by averaging over the distribution of electronic states based on the Ehrenfest ansatz:

$$M\ddot{\boldsymbol{R}}= -\boldsymbol{\nabla}_R[Tr\{\rho(t)H)/Tr(\rho(t)\}] \tag{2}$$

where $\rho(t)$ is obtained by solving the Liouville-von Neumann equation. In the above equation, R and M denote atomic coordinates and masses, respectively. The Mulliken population is calculated at each time step using the time propagated density matrix as $q_A = \mathrm{Tr_A}[\rho S]$, where $\mathrm{Tr_A}$ denotes the trace over orbitals centered on atom A.

We calculate absorption spectra for Au_{365} and Cu_{365} in the time domain by driving dipoles in the system with a delta kick, $E(t) = E_0\delta(t)\hat{e}$, with field amplitude E_0 and polarization $\hat{e} \in [x, y, z]$. $\delta(t)$ is the Dirac delta function. The Fourier transform of the induced dipole moment gives the absorption spectrum: $\sigma(\omega) = \frac{4\pi\omega}{3c}\Im[Tr\{(\mu(\omega) - \mu_0)/E_0\}]$, where $\mu(\omega)$ and μ_0 are the frequency-dependent and initial dipole moments, respectively. This approach provides absorption spectra which are identical to conventional calculations in the frequency domain when the field amplitude is smaller than the regime where non-linear optical processes become prevalent.

For the RT-TD-DFTB calculations, the time step for electronic structure evolution is set to be 0.0024 fs with the total number of steps set to 42,000. A laser pulse with a Gaussian envelope of the following form is used to drive the system, which induces the dissociation dynamics:

$$E(t) = E_0 e^{-(t-t_c)^2/T^2} \sin[\omega_0 t] \tag{3}$$

with a field amplitude E_0, a field maximum time t_c, pulse duration T, and driving frequency ω_0. Throughout, we use a Gaussian laser pulse polarized along the y-axis (denoted in illustrations of the geometries) with ω_0 = 2.5 eV and width parameter T = 14 fs. Figure S7 illustrates the temporal and frequency profile of the driving pulse.

Supporting Figures

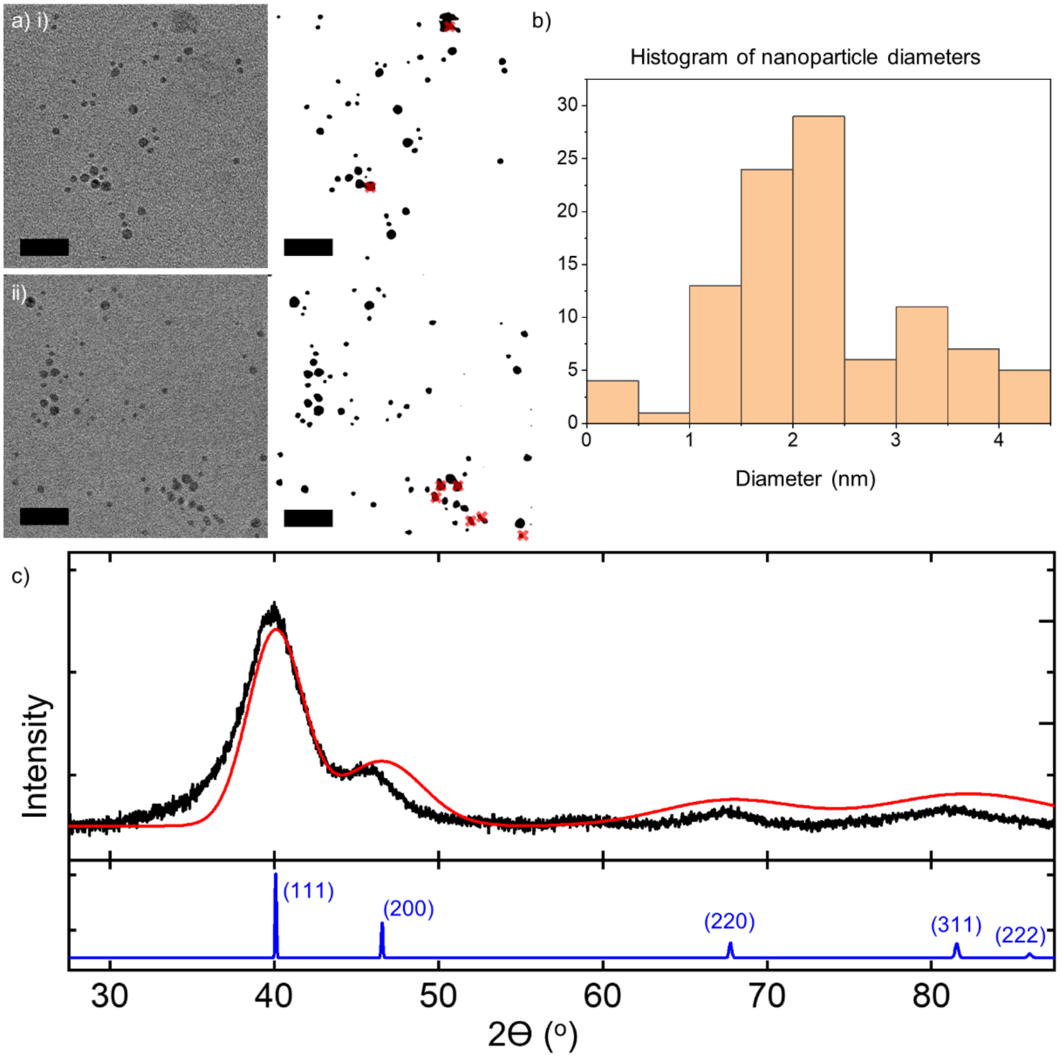

Figure S1| Structural characterization of as-synthesized Au–Cu nanoparticles. (a, b) Transmission electron microscopy (TEM) analysis of the nanoparticles. (a, left) Two low-magnification TEM images, (i) and (ii), showing many well-separated nanoparticles. For size analysis, these images were subjected to FFT-filtering and thresholding, resulting in the (a, right) panels on the right, which were then subjected to the 'Analyze Particles' tool in ImageJ to determine projected areas of well-separated nanoparticles. Several artifacts, nanoparticles on the edges of the image, and overlapping nanoparticles were excluded from this analysis, and are indicated by red 'X's on the thresholded images (a, right). From projected areas, diameters of the nanoparticles were calculated by assuming a spherical shape. All scale bars are 20 nm in length. (b) Histogram of diameters of 100 nanoparticles from the two provided images. (c) Powder X-ray diffraction (XRD) pattern of Au–Cu nanoparticles (black line, top plot), and a simulated pattern generated by a refinement of this pattern in PowderCell (red line, top plot). Refinement was most consistent with a face-centered cubic (FCC) lattice with a 3.93 Å lattice constant. For comparison, Au and Cu have FCC structures with lattice constants of 4.08 Å and 3.61 Å, respectively. Thus, using Vegard's law, the refinement suggests that the nanoparticles are comprised of an alloy of 68 mol % Au and 32 mol % Cu. The peaks in the patterns are assigned to specific reflections labeled by their Miller indices (blue sticks, bottom plot).

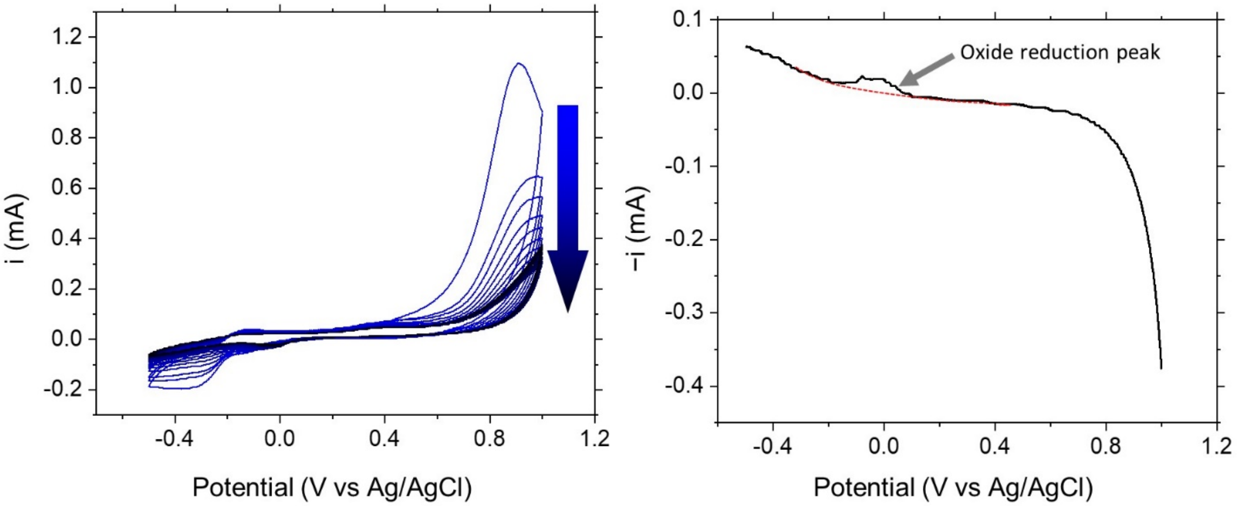

Figure S2| Electrochemical oxidation-reduction cycling and determination of electrochemically active surface area (ECSA). Left: CV scans of the Au–Cu NP coated-GCE in 1 M KOH acquired in the course of electrochemical oxidation–reduction cycling wherein the potential was swept from 0 V to 1.0 V and then 60 CV scans were conducted from 1.0 V to –0.5 V vs Ag/AgCl, sat. KCl and back at a scan rate of 50 mV/s. The first 22 scans recorded out of the 60 total scans are presented in this panel. Colors denote scan number, transitioning from blue to black with increasing scan number. Right: As shown by the final recorded cathodic sweep (shown with an inverted y-axis scale), a peak is seen in the CV scans around –0.1 V vs Ag/AgCl, sat. KCl. This peak corresponds to the reduction of metal oxide produced by oxidation of the surface of the nanoparticles at positive potentials of the scan. The area of this peak was used to determine the ECSA as described in the methods.

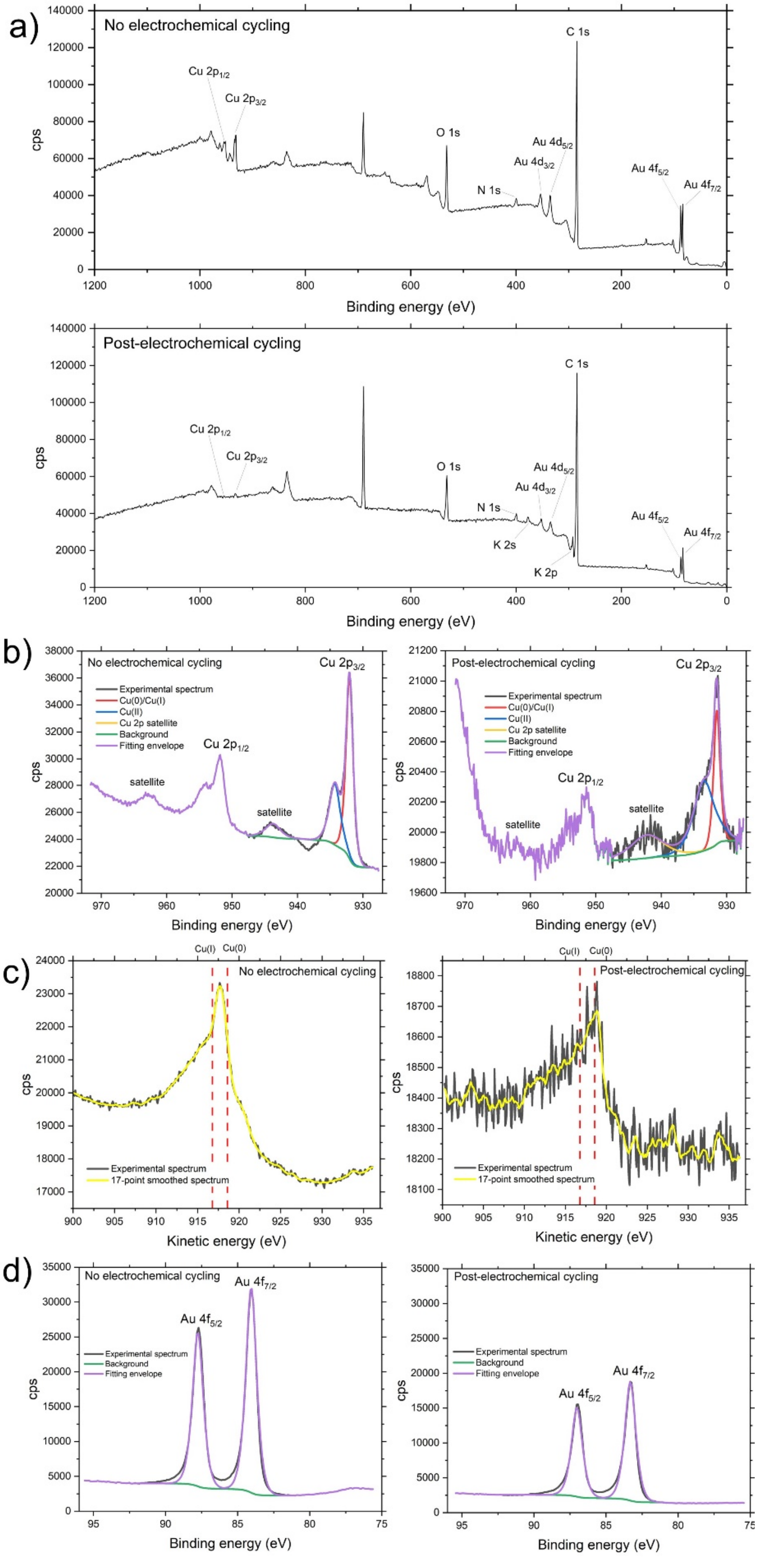

a)
No electrochemical cycling
Cu $2p_{1/2}$
Cu $2p_{3/2}$
O 1s
N 1s
Au $4d_{5/2}$
Au $4d_{3/2}$
C 1s
Au $4f_{5/2}$
Au $4f_{7/2}$
cps
Binding energy (eV)
Post-electrochemical cycling
K 2s
K 2p
b)
Experimental spectrum
Cu(0)/Cu(I)
Cu(II)
Cu 2p satellite
Background
Fitting envelope
satellite
c)
Cu(I)
Cu(0)
17-point smoothed spectrum
Kinetic energy (eV)
d)

Figure S3| XPS analysis of the chemical composition of the Au–Cu nanoparticles deposited on a GCP and subjected to either no further processing or subjected to electrochemical cycling steps. a) Survey spectrum indicating the presence of Au, Cu, C, and N with their relevant binding energy peaks labeled. C is from the oleylamine ligands as well as the glassy carbon substrate and adventitious carbon contamination.[S10] For the post-electrochemical cycling case, K likely originated from the electrolyte in which electrochemical cycling was performed. N is likely from oleylamine ligands and nitrogen content of glassy carbon.[S11] b) High-resolution spectrum in the Cu 2p region showing main Cu $2p_{3/2}$ and Cu $2p_{1/2}$ peaks at bindings energies of 932.1 eV and 951.8 eV corresponding to Cu(0) and/or Cu(I) (with further resolution possible only by an Auger spectrum in Cu LMM region), a main Cu $2p_{3/2}$ peak at 934.3 eV corresponding to Cu(II), and satellite peaks corresponding to Cu(II) or Cu(I) for the case of no electrochemical cycling. For the post-electrochemical-cycling case, these peaks appear at binding energies of 931.5 eV, 951.4 eV, and 933.4 eV, respectively. These individual components are shown by colored lines along with a fit of the experimental spectrum to a combination of these components and a background. The fit yields a residual standard deviation (STD) of 2.92 $(cps)^{1/2}$ for the case of no electrochemical cycling and 0.85 $(cps)^{1/2}$ for the post-electrochemical-cycling case. c) Auger spectra in the Cu LMM region for the cases of no electrochemical cycling and post-electrochemical-cycling. In each case, the spectrum is shown overlaid with a curve obtained by 17-point smoothing. The known Cu LMM peak positions[S12] for Cu(0) and Cu(I) at 918.6 eV and 916.8 eV, respectively, are shown by vertical dotted lines. The position of the experimental peak in the post-electrochemical-cycling case coincides more closely with Cu(0); however, the presence of Cu(I) cannot be fully ruled out. d) High-resolution spectra in the Au 4f region showing peaks at 84.0 eV and 87.7 eV for the case of no electrochemical cycling and 83.3 eV and 87.0 eV for the post-electrochemical-cycling case, which correspond respectively to Au $4f_{7/2}$ and Au $4f_{5/2}$ peaks for Au(0). The fit of the experimental spectrum to a combination of Au $4f_{7/2}$ and Au $4f_{5/2}$ peaks for Au(0) and a background is shown. The fit yields a residual STD of 15.21 $(cps)^{1/2}$ for the case of no electrochemical cycling and 17.15 $(cps)^{1/2}$ for the post-electrochemical-cycling case.

Thus, the nanoparticles subjected to electrochemical cycling are ascertained to contain Au and Cu in line with the as-synthesized nanoparticles. The composition is estimated by CasaXPS analysis to be 82 mol % Au and 18 mol % Cu on the basis of the integrated peak areas scaled by i) relative sensitivity factors (RSFs) for the Au $4f_{7/2}$ peak and the combination of the Cu $2p_{3/2}$ peaks (including the satellite peak) identified in panel (b), which were 9.58 and 16.73, respectively, according to CasaXPS analysis, and ii) mean free paths of the photoelectron, which were 1.778 nm for the Au $4f_{7/2}$ peak with a kinetic energy of ~1403 eV and 1.076 nm for the Cu $2p_{3/2}$ photoelectron with a kinetic energy of ~555 eV, according to literature.[S11] For comparison, the nanoparticles that had not been subjected to electrochemical cycling were found from XPS analysis to consist of 49 mol % Au and 51 mol % Cu. Thus, XPS analysis indicates the loss of some Cu by oxidative dissolution during electrochemical cycling. While the Au is primarily in the Au(0) state in the nanoparticles subjected to electrochemical cycling, the Cu appears to be in Cu(0) and Cu(II) states; however, Cu(I) cannot be fully ruled out. The nearly 2 eV difference between the main Cu $2p_{3/2}$ peaks suggests that Cu(II) is present as $Cu(OH)_2$ rather than CuO.[S13,S14]

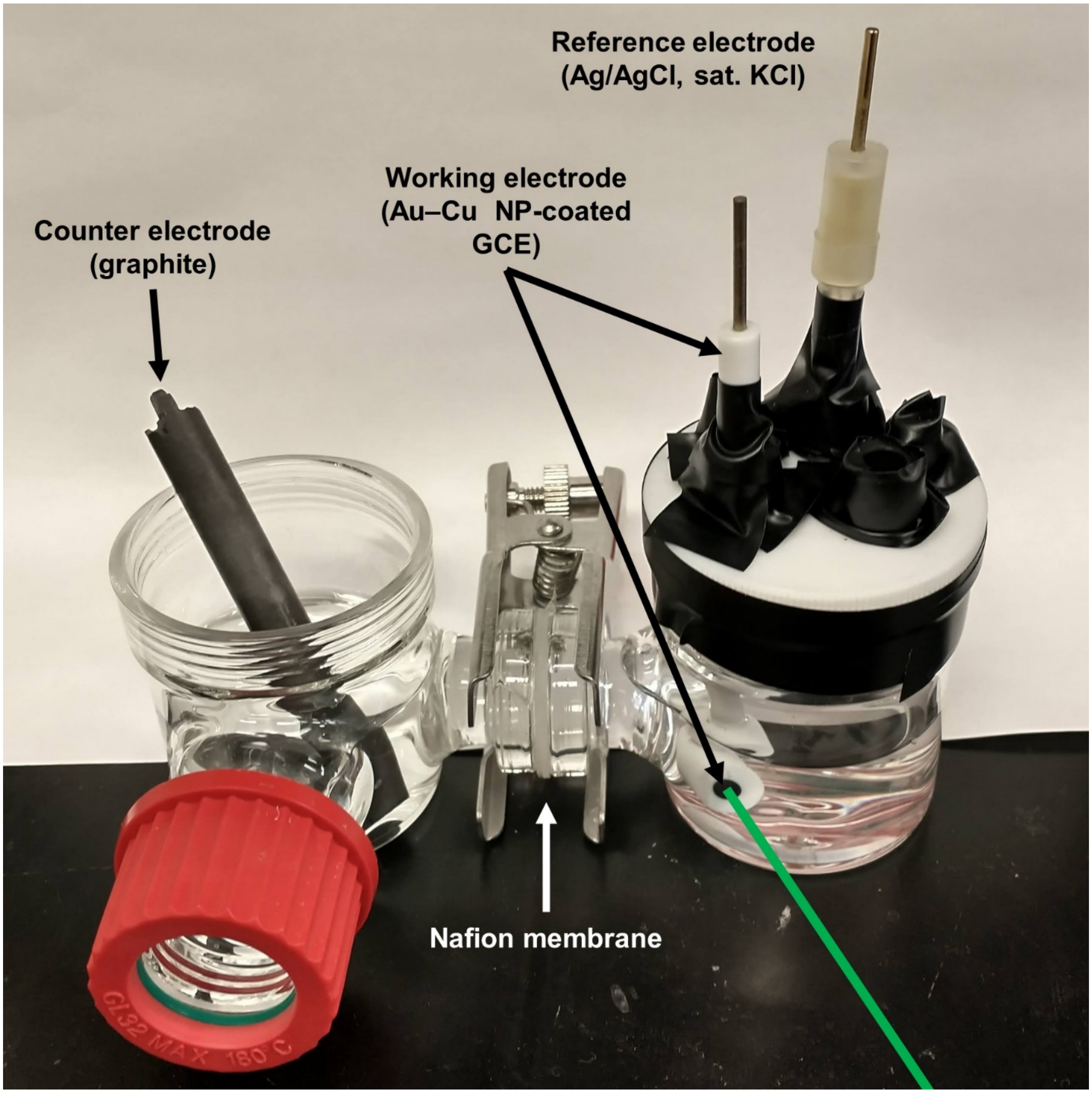

Figure S4| Photograph of electrochemical setup used for plasmon-assisted and dark electrocatalytic CO_2 reduction reaction (CO_2RR) experiments. A 532 nm laser beam is transmitted through a flat window in the right (cathodic) compartment of the glass cell and made incident on the exposed glassy carbon region of the working electrode.

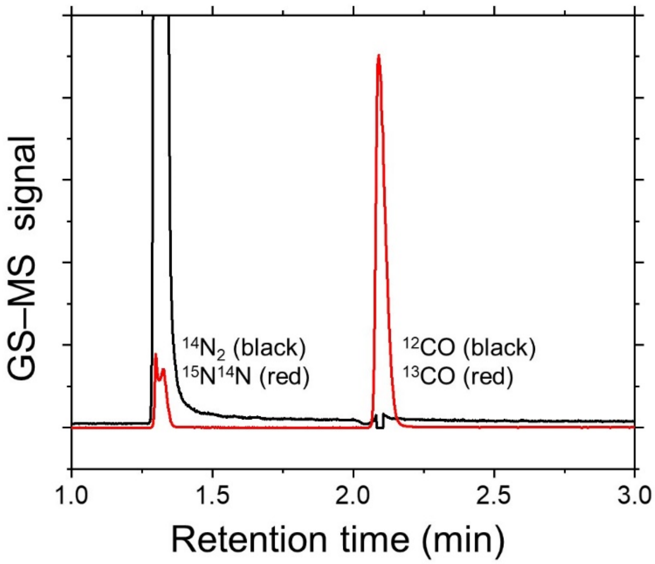

Figure S5| Confirmation of CO as a product of CO_2RR by ^{13}C-labeling. Gas chromatography–mass spectrometry (GC–MS) of $^{13}CO_2RR$ control. Chromatograms from GC–MS analysis of the headspace of the cathodic compartment of the electrochemical cell after a plasmon-assisted electrocatalytic CO_2RR experiment conducted at –1.54 V vs Ag/AgCl, sat. KCl with a Au–Cu nanoparticle-coated GCE and the catholyte saturated with $^{13}CO_2$. Chromatograms for m/z = 28 (black) and m/z = 29 (red) are plotted with the peaks assigned, as indicated by the labels. The large peak around a retention time of ~2.1 min in the m/z = 29 chromatogram corresponds to ^{13}CO; whereas no significant peak in the m/z = 28 chromatogram corresponding to ^{12}CO is observed. This verifies that the CO detected in the headspace following electrocatalysis is indeed produced from CO_2RR rather than from adventitious carbon sources or contaminants. Note that the peak at ~1.3 min in the m/z = 28 chromatogram corresponds to $^{14}N_2$ and is saturated due to the preponderance of atmospheric N_2. Secondly, the peak at ~1.3 min in the m/z = 29 chromatogram corresponds to $^{15}N^{14}N$, which has a non-negligible natural abundance in the atmosphere.

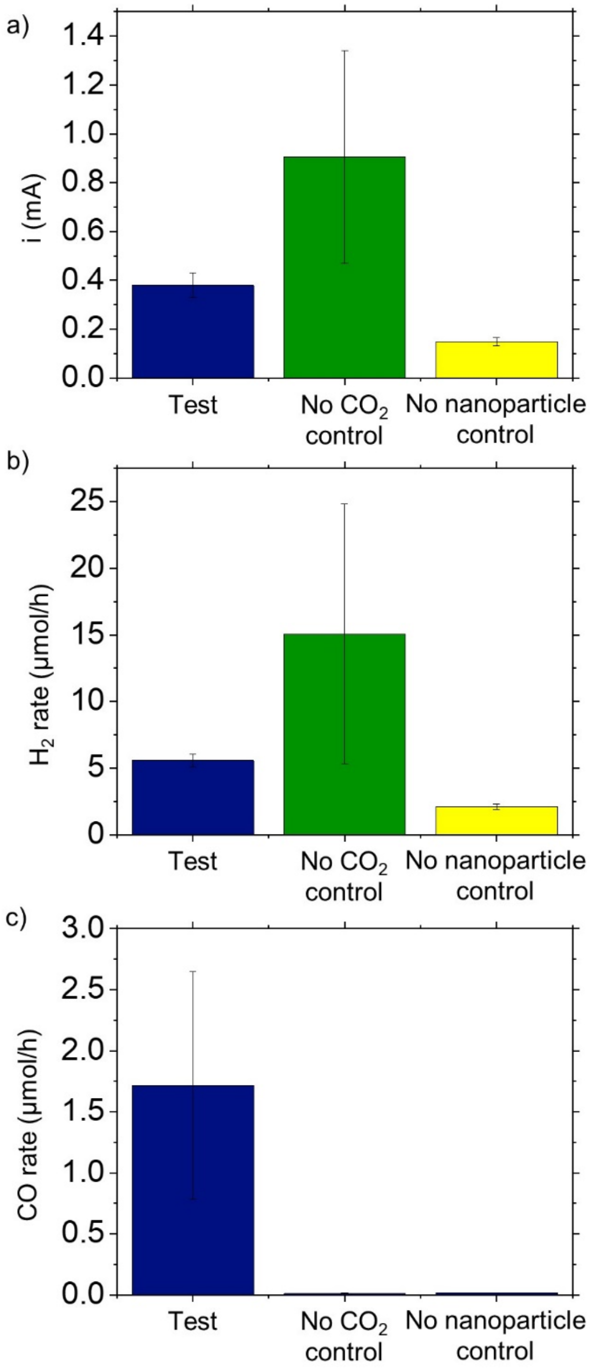

Figure S6| Results of control experiments performed without CO_2 and without Au–Cu nanoparticles. Bar graph of activity metrics: (a) average current, (b) rate of H_2 production, and (c) rate of CO production in the 8 h CA scan for a test run of plasmon-assisted electrocatalytic CO_2RR on Au–Cu nanoparticles at –1.54 V vs Ag/AgCl, sat. KCl (blue), control experiment without CO_2 saturation of the catholyte but with all other conditions maintained the same as the test run (green), and control experiment without Au–Cu nanoparticles deposited on the GCE but with all other conditions maintained the same as the test run (yellow). Each data point is the mean value from 3 identical trials on separate samples; the error bar shown represents the propagated standard error. The control experiments without Au–Cu alloy nanoparticles show a minor but non-zero rate of CO production, which is attributed to a low, intrinsic electrochemical activity of the GCE itself toward CO_2 reduction.[S15, S16] In the control experiments carried out without CO_2 saturation, we cannot rule out that the electrolyte contains a small concentration of CO_2 absorbed from the atmosphere, which may explain why we observe a minor but non-zero rate of CO production.

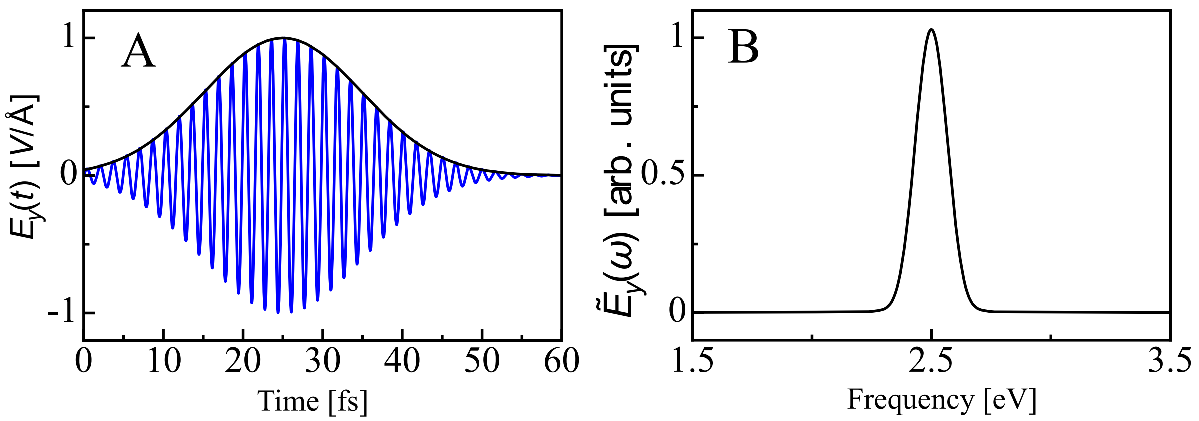

Figure S7| Profile of laser pulse used in RT-TD-DFTB simulations. (A) Time-dependent field $E(t)$ polarized along y-axis and (B) the normalized Fourier transform of $E(t)$ showing the frequency profile of the field. Here, central frequency ω_0 = 2.5 eV, pulse duration T = 14 fs, and field amplitude $E_0 = 1$ V/Å.

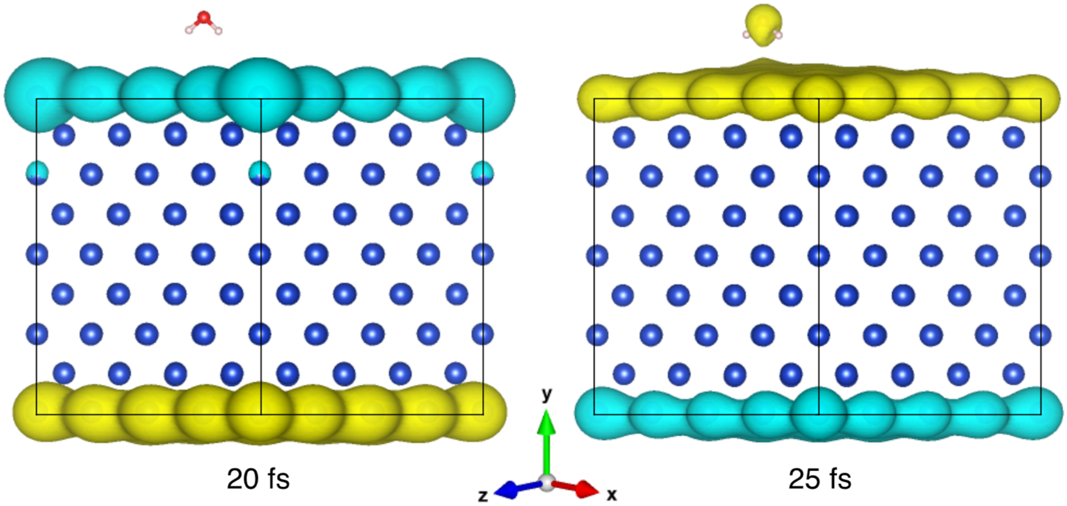

Figure S8| RT-TD-DFTB modeling of adsorbate photoactivation on Cu. Map of the difference in charge density (yellow for positive and cyan for negative) at 20 fs and 25 fs for $Cu_{365}H_2O$ with respect to the initial charge density at $t = 0$ when the system is driven by a Gaussian laser pulse with parameters $\omega_0 = 2.5$ eV, $T = 14$ fs, and $E_0 = 1.5$ V/Å. Cu atoms are shown in blue, H in light pink, and O in red.

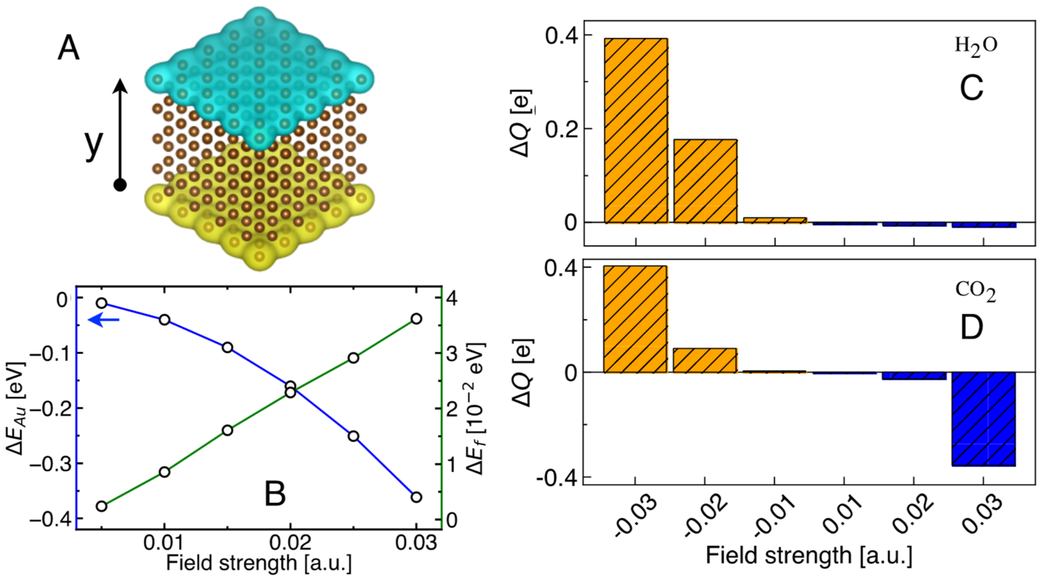

Figure S9| Effect of a static field on adsorbate activation modeled in DFTB. (A) Map of the difference in charge density (cyan for negative and yellow for positive) with respect to the charge density at zero static field for Au_{365} under a static electric field with an amplitude of 0.03 a.u. polarized along the y-axis, (B) Change in energy per Au atom (ΔE_{Au}) and change in Fermi energy (ΔE_f) as a function of the static field amplitude. Change in the charge ΔQ with respect to the charge at zero field for (C) H_2O and (D) CO_2 fragments at different field amplitudes.

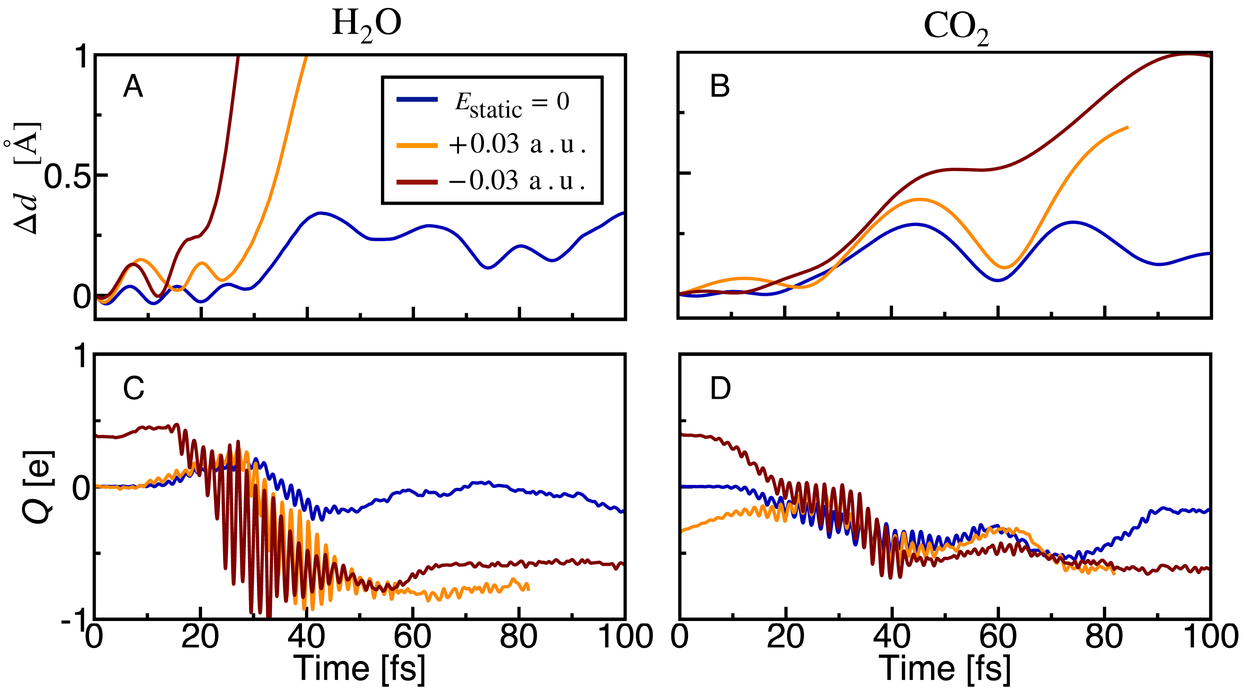

Figure S10| Effect of a static field on dissociation dynamics modeled in RT-TD-DFTB. Real-time trajectories (different replicas shown by different colors) for (A) change in O–H bond distance Δd and (B) C–O bond distance Δd in the $Au_{365}CO_2H_2O$ system under static fields of different amplitudes. The change is defined with respect to the initial value at $t = 0$ when a Gaussian laser pulse with parameters $\omega_0 = 2.5$ eV, $T = 14$ fs, and $E_0 = 1.5$ V/Å is applied to drive the system. The corresponding trajectories for (C) molecular charge Q on H_2O fragment and (D) molecular charge Q on CO_2 fragment, with the same color legend from panels A and B, respectively.

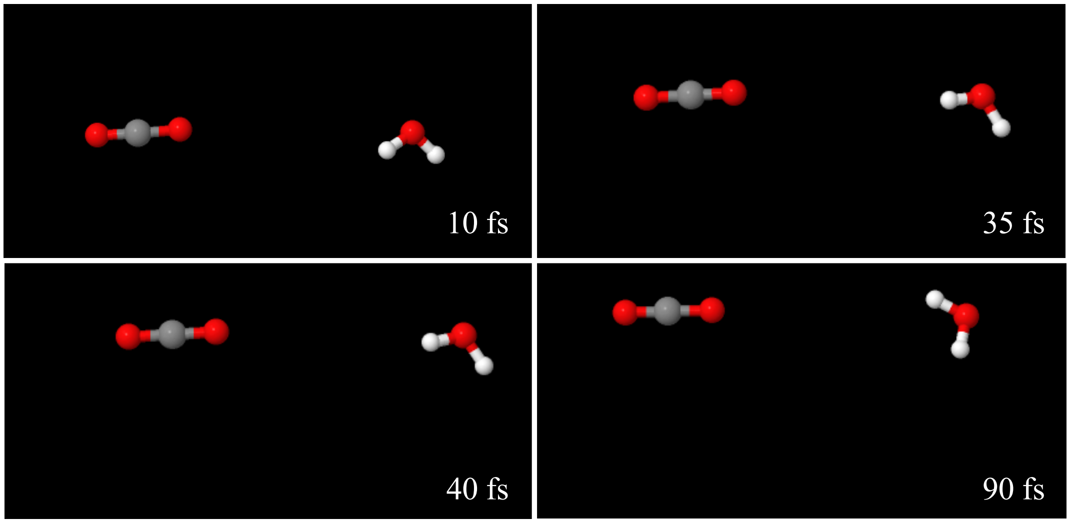

Figure S11| Dissociation dynamics for isolated molecules (without the nanoparticle) interacting with a laser pulse. Snapshots of geometries of H_2O and CO_2 for four different times along a trajectory. At $t = 0$ s, a Gaussian laser pulse extending between [0, 50] fs, a central frequency $\omega_0 = 2.5$ eV, and a field amplitude of 2 V/Å was applied. No molecular dissociation is observed.

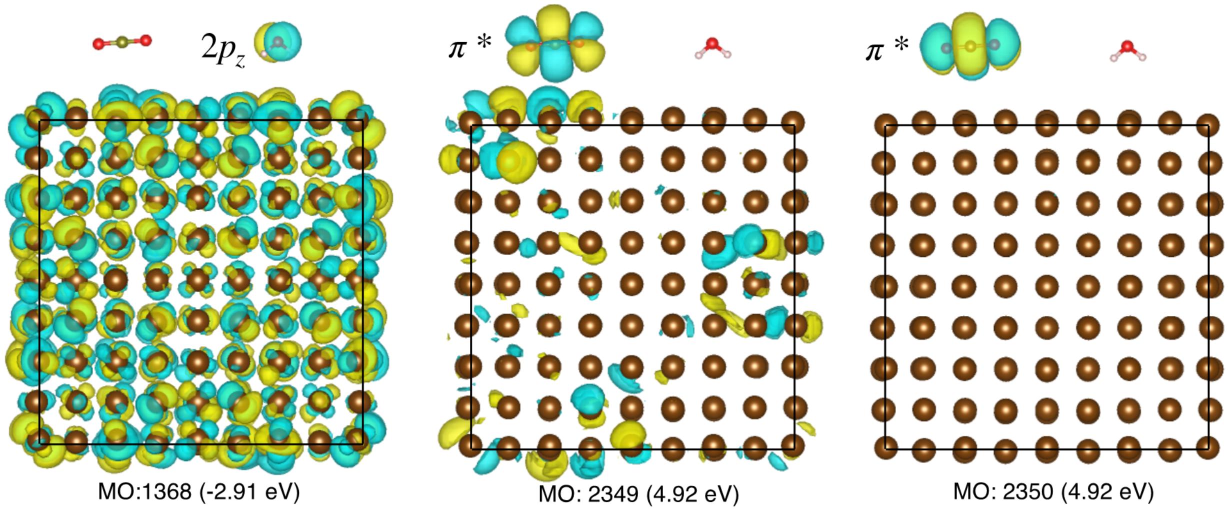

Figure S12| Orbitals involved in charge transfer. Visualization of the wavefunctions for Kohn-Sham orbitals of the $Au_{365}CO_2H_2O$ system with significant localization of wavefunction amplitude on the molecule and thereby which are likely involved in Au-to-molecule charge transfer: (left) the highest-occupied molecular orbital (HOMO), of $2p_z$ character, of H_2O and (middle and right) unoccupied orbitals of CO_2. Cyan and yellow represent opposite phases of the wavefunction. Orbital indices are shown under each plot along with the orbital energy relative to the Fermi energy in parentheses. For this analysis, Kohn-Sham orbitals of the optimized structure of the $Au_{365}CO_2H_2O$ system were calculated by the time-dependent density-functional tight-binding (TD-DFTB) method. Starting from the Fermi energy, we scanned across orbitals energies both above and below the Fermi energy to identify the closest-lying orbitals with significant wavefunction amplitude localized on H_2O or CO_2. Wavefunctions of these orbitals were plotted using VESTA and DFTB+/Waveplot program and presented here.

Supporting Table

Table S1: FEs for the experiments presented in Fig. 3.

Experimental Conditions	CO FE (%)	H_2 FE (%)	Sum of CO and H_2 FEs (%)
–1.44 V vs Ag/AgCl, sat. KCl plasmonic	10 (± 4)	95 (± 31)	105 (± 31)
–1.44 V vs Ag/AgCl, sat. KCl dark	37 (± 4)	47 (± 25)	84 (± 25)
–1.54 V vs Ag/AgCl, sat. KCl plasmonic	24 (± 14)	79 (± 14)	103 (± 20)
–1.54 V vs Ag/AgCl, sat. KCl dark	37 (± 13)	48 (± 15)	85 (± 19)

Each CO and H_2 FE listed in the second and third columns is a mean value across three trials on separate samples; the propagated standard error is listed in parentheses. All tabulated values are in terms of percentages. The rightmost column provides a simple sum of the corresponding CO FE and H_2 FE; the standard error obtained by error propagation for a sum is reported in parentheses. We find that the sum of CO and H_2 FEs is ~100% under plasmonic excitation conditions, whereas it is lower under dark conditions. It is possible that other minor product/s account for the remaining balance under dark conditions. Formate is a likely candidate based on Yang and coworkers' findings on Au–Cu nanoparticles,[S17] which would imply that plasmonic excitation suppresses formate formation alongside the suppression of the CO production pathway.

Supporting References

(S1) Chen, W.; Yu, R.; Li, L.; Wang, A.; Peng, Q.; Li, Y. A Seed-Based Diffusion Route to Monodisperse Intermetallic CuAu Nanocrystals. *Angew. Chem. Int. Ed.* **2010**, *49* (16), 2917–2921.

(S2) Peng, S.; Lee, Y.; Wang, C.; Yin, H.; Dai, S.; Sun, S. A Facile Synthesis of Monodisperse Au Nanoparticles and Their Catalysis of CO Oxidation. *Nano Res.* **2008**, *1* (3), 229–234.

(S3) Wang, J.; Heo, J.; Chen, C.; Wilson, A. J.; Jain, P. K. Ammonia Oxidation Enhanced by Photopotential Generated by Plasmonic Excitation of a Bimetallic Electrocatalyst. *Angew. Chem. Int. Ed.* **2020**, *59* (42), 18430–18434.

(S4) Contreras, E.; Nixon, R.; Litts, C.; Zhang, W.; Alcorn, F. M.; Jain, P. K. Plasmon-Assisted Ammonia Electrosynthesis. *J. Am. Chem. Soc.* **2022**, *144* (24), 10743–10751.

(S5) Hansen, S. V. F.; Ulven, T. Oxalyl Chloride as a Practical Carbon Monoxide Source for Carbonylation Reactions. *Org. Lett.* **2015**, *17* (11), 2832–2835.

(S6) Hourahine, B.; Aradi, B.; Blum, V.; Bonafé, F.; Buccheri, A.; Camacho, C.; Cevallos, C.; Deshaye, M. Y.; Dumitric, T.; Dominguez, A.; Ehlert, S.; Elstner, M.; Van Der Heide, T.; Hermann, J.; Irle, S.; Kranz, J. J.; Köhler, C.; Kowalczyk, T.; Kubař, T.; Lee, I. S.; Lutsker, V.; Maurer, R. J.; Min, S. K.; Mitchell, I.; Negre, C.; Niehaus, T. A.; Niklasson, A. M. N.; Page, A. J.; Pecchia, A.; Penazzi, G.; Persson, M. P.; Řezáč, J.; Sánchez, C. G.; Sternberg, M.; Stöhr, M.; Stuckenberg, F.; Tkatchenko, A.; Yu, V. W. Z.; Frauenheim, T. DFTB+, A Software Package for Efficient Approximate Density Functional Theory Based Atomistic Simulations. *J. Chem. Phys.* **2020**, *152,* 124101.

(S7) Bonafé, F. P.; Aradi, B.; Hourahine, B.; Medrano, C. R.; Hernández, F. J.; Frauenheim, T.; Sánchez, C. G. A Real-Time Time-Dependent Density Functional Tight-Binding Implementation for Semiclassical Excited State Electron-Nuclear Dynamics and Pump-Probe Spectroscopy Simulations. *J. Chem. Theory Comput.* **2020**, *16,* 4454–4469.

(S8) Sánchez, C. G.; Berdaki, M. Plasmon-Induced Hot Carriers: An Atomistic Perspective of the First Tens of Femtoseconds. *J. Phys. Chem. C* **2022**, *126*, 10015-10023.

(S9) Giri, S. K.; Schatz, G. C. Photodissociation of H_2 on Ag and Au Nanoparticles: Effect of Size and Plasmon versus Interband Transitions on Threshold Intensities for Dissociation. *J. Phys. Chem. C* **2023** *127*, 4115-4123.

(S10) Sinha, S.; Mukherjee, M. A Study of Adventitious Contamination Layers on Technically Important Substrates by Photoemission and NEXAFS Spectroscopies. *Vacuum.* **2018**, *148*, 48–53.

(S11) Dekanski, A.; Stevanović, J.; Stevanović, R.; Nikolić, B. Ž.; Jovanović, V. M. Glassy Carbon Electrodes: I. Characterization and Electrochemical Activation. *Carbon* **2001**, *39,* 1195–1205.

(S12) *Thermo Fisher Cu X-ray photoelectron spectra.*

https://www.thermofisher.com/us/en/home/materials-science/learning-center/periodic-table/transition-metal/copper.html (accessed 2024-01-04).

(S13) Powell, C. J.; Jablonski, A. Evaluation of Calculated and Measured Electron Inelastic Mean Free Paths Near Solid Surfaces. *J. Phys. Chem. Ref. Data* **1999**, *28*, 19–62.

(S14) Biesinger, M. C. Advanced Analysis of Copper X-Ray Photoelectron Spectra. *Surf. Interface Anal.* **2017**, *49*, 1325–1334.

(S15) Christensen, P.A.; Hamnett, A.; Muir, A.V.G.; Freeman, N.A. CO_2 Reduction at Platinum, Gold And Glassy Carbon Electrodes in Acetonitrile: An In-Situ FTIR Study, *J. Electroanal. Chem. Interf. Electrochem.* **1990**, *288*, 197–215.

(S16) Hara, K.; Kudo, A.; Sakata, T. Electrochemical CO_2 Reduction on a Glassy Carbon Electrode Under High Pressure, *J. Electroanal. Chem.* **1997**, 421, 1–4.

(S17) Kim, D.; Resasco, J.; Yu, Y.; Asiri, A. M.; Yang, P. Synergistic Geometric and Electronic Effects for Electrochemical Reduction of Carbon Dioxide Using Gold-Copper Bimetallic Nanoparticles. *Nat. Commun.* **2014**, *5*, 4948.
